# Post-Wildfire Indoor Pollution in WUI Areas following the 2025 Los Angeles Fires. Part I. Establishing Baseline Contaminant Levels Prior to Home Reoccupation

**DOI:** 10.1021/acsestair.5c00281

**Published:** 2025-12-19

**Authors:** Ehsan Goftari, Jose Rivera Carias, London Fulford, Hanyang Li

**Affiliations:** † Department of Civil, Construction, and Environmental Engineering, 7117 San Diego State University, San Diego, California 92182, United States; ‡ Department of Mechanical and Aerospace Engineering, University of California, San Diego, La Jolla, California 92037, United States

**Keywords:** wildland−urban interface, wildfires, air toxics, indoor air pollution, Palisades and Eaton fires

## Abstract

Wildland–urban interface (WUI) fires pose unique environmental pollution challenges due to the combustion of both natural vegetation and synthetic building materials. Following the 2025 Palisades and Eaton wildfires in Los Angeles, we conducted a field study to characterize indoor air quality and surface contamination in 19 homes before reoccupancy. Indoor PM_2.5_ and PM_10_ concentrations averaged 3.45 and 31.66 μg/m^3^, respectively, with several homes showing indoor-to-outdoor (I/O) ratios of >1 (particularly for PM_10_) compared to typical I/O values of 0.45–0.8 in residences, indicating persistent indoor particle reservoirs. Depending on the air-exchange rate, elevated indoor PM levels in noncleaned homes may arise from two contrasting mechanisms: low ventilation that traps resuspended fire residues triggered by movements during sampling and high ventilation that induces turbulence and disturbs heavily contaminated entry zones. Regression analysis suggested that proximity to the fire, absence of air purifiers, use of non-HEPA vacuums, and open windows during the fire significantly increased indoor PM levels, explaining 73% (PM_10_) and 86% (PM_2.5_) of the variation across homes. Airborne metal concentrations were below health-based thresholds; however, surface wipe samples revealed widespread contamination, with potassium, magnesium, aluminum, and iron frequently exceeding 1000 μg/ft^2^, and detectable levels of zinc, copper, and manganese in many homes. Lead concentrations exceeded the EPA’s dust clearance levels in multiple homes, especially on window sills and entry floors. Our findings highlight that while airborne risks may subside within weeks after the fire, indoor surfaces can retain fire-related pollutants, presenting ongoing exposure risks even 2 months after the fire.

## Graphical abstract

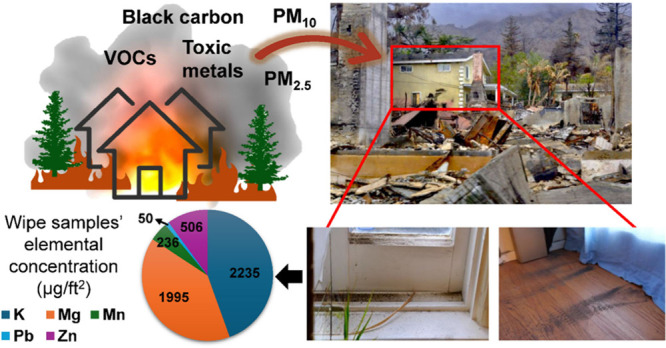

## 1 Introduction

As urban areas continue to expand, more people are living in or near wildland regions, known as the wildland–urban interface (WUI). This development has brought communities closer to nature but has also significantly increased their exposure to WUI fire hazards. As a result, the risks of structural damage, economic loss, and adverse health impacts have risen dramatically. Currently, more than 70000 communities (home to nearly 160 million people) are at risk, with WUI areas expanding by approximately 2 million acres annually.^1^ Historically, 7 of the 15 largest fire-related disasters in the U.S. have been WUI fires.^2^ Recent devastating WUI fires include the 2023 Maui Wildfire in Hawaii, the 2021 Marshall Fire in Colorado, the 2016 Gatlinburg Fires in Tennessee, and the 2014 Bastrop County Complex in Texas. Most recently, in January 2025, the Palisades and Eaton fires, fueled by dry and strong Santa Ana winds, caused 30 deaths, forced over 100000 people to evacuate, and led to the destruction of 6822 and 9413 structures, respectively.^3,4^ Moreover, air quality significantly deteriorated across the region, with elevated levels of particulate matter (PM) and toxic metal particulates (e.g., Pb), as well as benzene, toluene, ethylbenzene, and xylenes (BTEX), particularly impacting communities in Malibu and Altadena.^5–7^ In response, the California Air Resources Board (CARB), the South Coast Air Quality Monitoring District (SCAQMD), and collaborative research initiatives [such as the Postfire airborne Hazard Observation Environmental Network for Integrated Xposure-monitoring (PHOENIX) project and the LA Fire HEALTH Study] launched comprehensive stationary and mobile air quality measurements in the fire-affected areas to investigate pollutant dynamics and assess long-term public health impacts.^8–11^

Unlike wildland fires that primarily burn vegetation, WUI fires ignite a combination of natural and synthetic materials, including plastics, treated wood, electronics, and household furnishings, which are present in various quantities and densities. The combustion of these materials releases a complex mixture of air pollutants, including volatile organic compounds (VOCs), polycyclic aromatic hydrocarbons (PAHs), and other semi-VOCs, metal compounds, and carbon-containing particulates.^12^ These outdoor air pollutants produced during WUI fire events can infiltrate surviving homes through windows, doors, cracks, and HVAC systems, negatively impacting indoor air quality (IAQ).^13,14^

The health risks of WUI fire emissions extend beyond the immediate fire events, as many residents return to their homes within hours or days after evacuation orders are lifted, often unaware of the hazardous pollutants that remain indoors.^15^ This is particularly concerning given that individuals spend the majority of their time indoors, especially in their homes, where long-term exposure to these pollutants can significantly increase the risk of cardiovascular diseases, respiratory problems, and neurodegenerative disorders.^16–18^ In the absence of readily accessible indoor air monitoring data, residents often rely on sensory cues (such as the distinct odors of smoke, which can range from a metallic smell of burned electronics or rubber to a woody scent from the combustion of organic materials) to judge air quality.^19^ These methods are subjective and unreliable, making them inadequate for guiding personal protection and exposure reduction. Thus, there is a need to move beyond subjective assessments of air quality and develop reliable methods to characterize indoor pollutants and evaluate their long-term health impacts following WUI fire events.

While some efforts have been made to monitor ambient air quality affected by WUI fires, IAQ remains relatively understudied. Kirk et al. (2018) conducted IAQ measurements in two homes in the Pacific Northwest during the summer 2015 wildfire season and found that indoor PM_2.5_ concentrations reached an average of 15 μg/m^3^ during active fire periods.^20^ A more recent study in Western Montana monitored 20 homes throughout the 2022 wildfire season and found that indoor PM_2.5_ levels rose substantially during smoke events.^21^ Specifically, the mean indoor PM_2.5_ during wildfire periods was 15.9 μg/m^3^, compared to 5.6 μg/m^3^ during nonwildfire periods (i.e., near 3-fold increase). Although protective actions, such as closing windows and using portable air cleaners, can mitigate indoor PM_2.5_ exposure during wildfire events, many homes still exhibited elevated indoor PM_2.5_ levels. In a large-scale California study, Liang et al. (2021) reported that indoor PM_2.5_ concentrations during fire events in over 1400 buildings show nearly tripled values compared to nonfire days.^13^ The elevated concentrations came back to background levels approximately two to 7 days after the wildfire plume had subsided. The study also found that newer buildings and the use of air filtration during fires can mitigate indoor exposure by 18% and 73%, respectively.

Some studies have shown that the retention of fire-related pollutants in indoor environments varies significantly by compound class. For example, based on indoor dust collected 8 days after the Marshall Fire, Silberstein et al. (2023) reported that PAHs concentrations in smoke-affected homes reached a median of 1859.3 ng/g, representing a significant enhancement over background levels.^22^ In contrast, Kohl et al. (2019) found limited persistence of wildfire-derived PAHs in house dust 14 months after the Fort McMurray fire, with concentrations generally lower than those in unaffected urban areas.^23^ This contrast highlights that while PAHs can remain embedded in indoor dust shortly after a fire, their long-term persistence appears to decrease over time. Meanwhile, studies by Dresser et al. (2025) and Li et al. (2023) showed that although airborne VOC levels declined rapidly within hours after smoke exposure, VOCs continued to off-gas from indoor materials for weeks, indicating the importance of surface reservoirs.^24,25^ In comparison, the elevated PM_2.5_ concentrations in Marshall fire-affected homes were found to decline to near-background levels within weeks, and only a few metals (As, Cr, Cu, Pb, and Zn) exhibited moderate enrichment (enrichment factors of 2–5) in indoor dust samples, likely due to pre-existing urban dust and rapid dispersal of fire-emitted particles.^22^ Similarly, the trace metal concentrations in house dust 14 months after the Fort McMurray fire were similar to other Canadian cities, with only As showing a modest 62% elevation in neighborhoods where buildings had burned.^23^

To date, most studies on IAQ following WUI fires have measured conditions at isolated time points (such as during the fire, within days or weeks afterward, or over a year later), without capturing the temporal progression of indoor environmental conditions during recovery, reoccupation, and renewed human activity. To address this gap, we initiated a multiphase field study focused on homes impacted by the 2025 Palisades and Eaton fires. This article presents the first publication from the study, aimed at establishing baseline levels of airborne pollutants and surface contamination inside homes before reentry. Subsequent phases of this study will examine air pollutant dynamics throughout the postfire recovery timeline during re-entry and reconstruction, as well as the long-term persistence of indoor contamination. Collectively, this work will offer foundational data to support evidence-based remediation guidance and inform safe reentry strategies for fire-impacted communities.

## 2 Methods

### 2.1 Volunteer Recruitment, Home Selection, and Sampling

We responded after the fires by sending out a questionnaire on February 11, 2025, to recruit participants from fire-affected areas for our study. The questionnaire included questions such as extent of fire damage, timing of reentry, power availability, cleanup and reconstruction plans, and consent for on-site sampling. The full questionnaire is provided in the Supporting Information. As of February 20th, 262 residents expressed interest in participating, with 167 reporting noticeable smoke odors indoors and 142 indicating that accumulated ash had not yet been removed. Additionally, many residents reported respiratory issues, eye irritation, and concerns about long-term exposure to residual ashes. Some noted visible soot accumulation on indoor surfaces, while others experienced headaches and worsened allergy symptoms since returning home. Power outages and incomplete remediation efforts have further compounded these challenges, leaving many homeowners uncertain about the safety of their indoor environment.

On the basis of the responses we received from homeowners, we categorized the houses into three distinct groups according to the fire impact and smoke exposure, as shown in Figure 1. The green category includes homes that met all three criteria: (1) experienced some level of structural damage from the fire, (2) had not been cleaned, and (3) exhibited noticeable indoor smoke odors. The blue category includes homes that met two partial criteria: (1) had not undergone any cleaning and (2) either visible fire damage or noticeable indoor smoke odors were present. Lastly, the gray category represents homes lacking sufficient criteria to be classified as green or blue. To capture a diverse range of sampling environmental conditions, we selected a total of 16 homes in Altadena and Pasadena, mainly from the green and blue categories, with a limited number from the less-affected gray category. Additionally, three homes from Malibu, affected by the Palisades fire, were included, resulting in a final sample set of 19 homes. Prior to finalizing our sampling plan, we confirmed with homeowners that no smoking occurs inside these residences, thereby ensuring that our measurements were not influenced by lingering tobacco smoke contamination.

**Figure 1:**
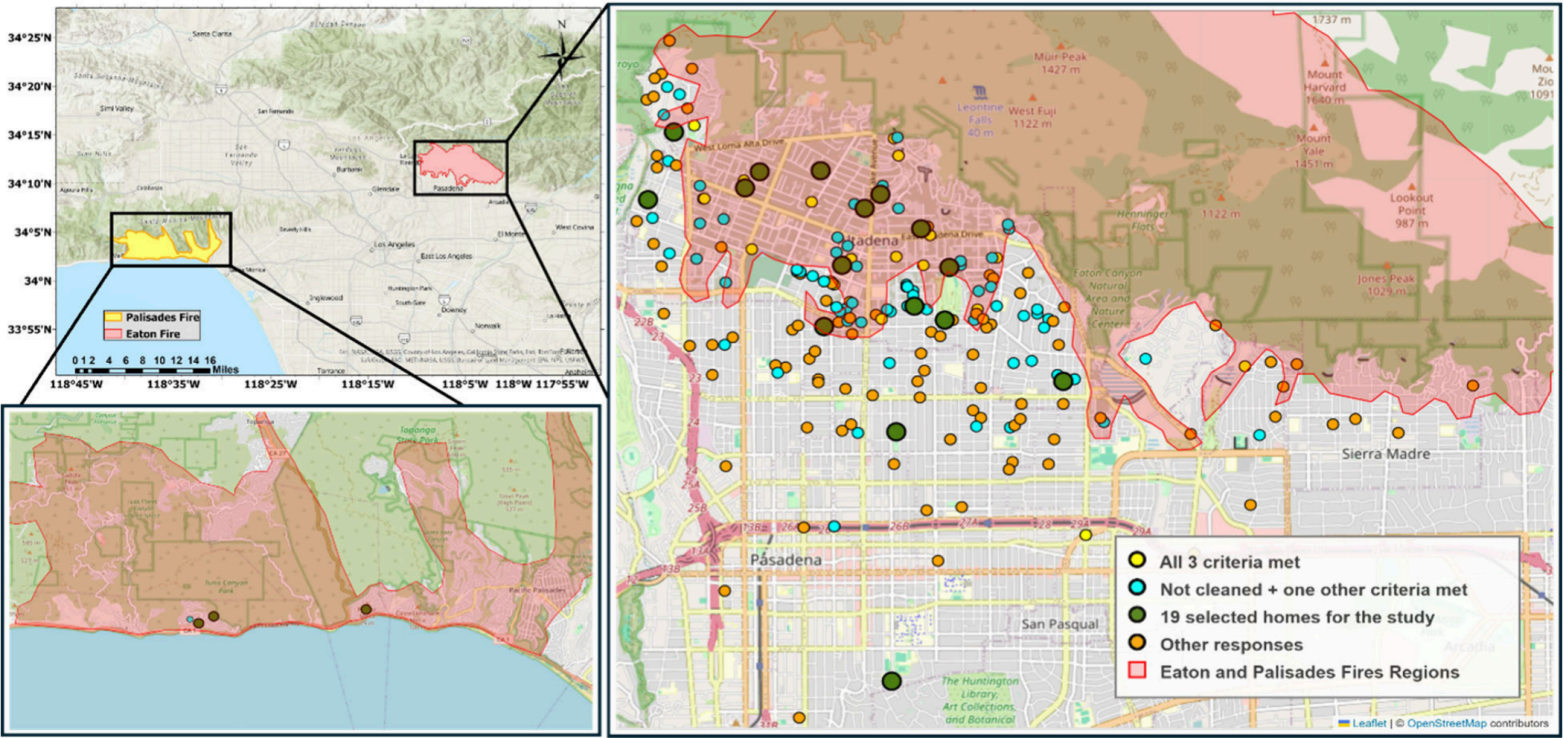
Detailed view of the Eaton fire region with the categorization of homes selected for this study. Exact home locations are generalized to protect resident privacy. Assigned IDs to each sampled household can be found in Figure S1.

Following coordination with the selected homeowners, our monitoring campaign began on March 6th, 2025 and spanned six consecutive days, including 5 days of indoor measurements in the Eaton Fire region and a final day in the Palisades Fire region (March 11, 2025). Each day, we visited and sampled three to four homes sequentially, allowing for detailed assessments while maximizing spatial coverage across the affected areas. During a 3-h sampling session at each home, we conducted indoor air quality measurements at a height near the breathing zone (∼1.5 m) in one of the commonly used spaces, such as the living or family room. The sampling cart was positioned at the center of the room, away from walls, windows, and HVAC vents, and windows and doors were kept closed to ensure consistent measurement conditions across homes. Alongside air quality sampling, we collected surface wipe samples from various contaminated indoor surfaces (see Section 2.3 for more details on the dust sample collection). Instrument setup and surface wipe sample collection were typically completed within the first 15 min after entry. After the setup and collection of wipe samples, indoor activities were minimized, and only one investigator remained inside to monitor the status and performance of the air quality instruments. Measurements were accompanied by contextual information, including ventilation status, use of air purifiers, and surface characteristics (e.g., carpet versus hard flooring). At each home, we also took photographs to document significant ash and soot contamination, structural damage, and surfaces from which we took wipe samples. Additionally, we conducted interviews with homeowners to gather self-reported information such as the building age and structural material, window types, recent cleaning activities, and future remediation plans.

To protect participant privacy, we will use anonymized identifiers (e.g., Homes 1306, 2306, 1307, etc.) when referring to the 19 sampled homes throughout this paper. These identifiers were assigned logically to reflect the study region and sampling order without revealing specific addresses. Among these identifiers, the three sampled homes in Malibu begin with the prefix “M”. One residence, Home 2307, was relatively larger compared to other sampling homes, so we sampled two distinct areas separately and assigned them identifiers Home 2307-1 and Home 2307-2 to better capture intrahome spatial heterogeneity. Among all sampled homes, Home 3307 was less affected by the fire due to its distance from the burn zone and had been professionally cleaned prior to our visit; it is used as a benchmark to represent a remediated indoor environment in the study for comparison with more impacted homes.

### 2.2 Measurement Instruments

The indoor PM_2.5_ and PM_10_ concentrations were measured using a real-time PM monitor (MODULAIR, QuantAQ Inc., Boston, MA) with a 1 min temporal resolution and up to 2000 μg/m^3^ measurement range (uncertainty: ∼10% for PM_2.5_ and 25% for PM_10_). The sensor integrates nephelometry, which measures the total light scattering from a particle population across multiple angles, and an optical particle counter (OPC), which counts and sizes individual particles as they pass through a laser beam, to determine particle mass concentration.^26,27^

Indoor black carbon (BC) concentrations were measured using a microAeth MA200 aethalometer (AethLabs, San Francisco, CA), which has a limit of detection (LOD) of 30 ng/m^3^ and a 5-min sampling resolution.^28^ This instrument collects BC particles on a PTFE tape and simultaneously passes light at multiple wavelengths (ultraviolet, blue, green, red, and infrared) through the tape. As the BC particles accumulate on the tape, they absorb more light, resulting in the attenuation of transmitted light intensity. The attenuated light is then measured through an optical detector, which is then used to quantify the concentration of carbon-containing particles based on changes in the light intensity at each wavelength. In this study, we used the concentrations at the infrared band, as it is found to be the dominant wavelength absorbed by BC.^29,30^

Total VOC (TVOC) concentrations were measured using the HVX501 Hand-held VOC Meter^31^ (Hal Technology, Pomona, CA), which features a 1-min sampling resolution, a LOD of 10 ppb, and a measurement range up to 20 ppm. This instrument is equipped with a photoionization detector (PID) sensor that uses an ultraviolet lamp to ionize VOC molecules as they pass through the sensor chamber. The resulting ions are attracted to a charged electrode, generating a small electrical current proportional to the VOC concentration. Since the PID responds collectively to a broad range of VOCs without distinguishing individual compounds, the sensor provides an aggregate measure of TVOCs rather than speciated data. Compared to other low-cost VOC sensors such as metal oxide semiconductor (MOS) or electrochemical sensors, PID-based sensors offer higher sensitivity and better linearity, making them more suitable for accurate real-time VOC monitoring.^32,33^

Toxic metal particulate concentrations were measured using Toxic-metal Aerosol Real-Time Analyzer (TARTA), Version 2.0, developed by San Diego State University and the University of California, Davis.^34–37^ In this study, TARTA was configured to sample total suspended particles (TSP) without a size-selective inlet. TARTA employs spark-induced breakdown spectroscopy (SIBS) to quantify airborne particulate metal concentrations in near real time. In this setup, aerosol particles are collected on the surface of an electrode over a 30-min sampling period. A high-voltage spark (∼5 kV) is then applied to ablate the deposited particles, causing the ionized materials to emit light as they return to their ground states. The emitted light is captured by an optical spectrometer, and metal concentrations are quantified based on the intensity of element-specific emission wavelengths (see Section 2.4 for more details). Compared to TARTA 1.0, this new version features a bigger ground electrode (⌀ 2.4 mm), no optical lens (less alignment effort needed), lower weights (∼6 lbs.), smaller dimensions (8 in. × 10 in. footprint), and better LODs.^38^

All four instruments used in this study for indoor measurements were portable and battery-powered, enabling deployment in postfire environments where many homes had not yet regained power due to damage to local infrastructure. To facilitate mobility and setup, we mounted the instruments and three power banks on a lightweight, wheeled cart (37.5 × 35 × 62.5 cm) to ensure compactness, easy maneuverability between rooms and areas, and up to 9 h of continuous sampling per day without the need for external power sources (Figure S2a).

The outdoor PM_2.5_ and PM_10_ concentrations were extracted from the PHOENIX network, a collaborative initiative by the California Institute of Technology and local community members in Altadena and Pasadena.^10^ The PHOENIX network consists of 28 monitoring stations deployed across the Eaton Fire area, each equipped with a QUANTAQ PM sensor (the same sensor as used in our indoor sampling) that provides 1 min resolution data. As of our sampling date, 11 of the 28 sensors had already been installed and therefore used in our estimation of the outdoor PM concentrations corresponding to each indoor sampling location and time period.

### 2.3 Dust Sampling and Analytical Method

We collected a total of 30 surface wipe samples, with at least one sample per home, from a variety of indoor surfaces, including carpets, floors, counters, and tables, specifically targeting areas most visibly affected by ash and soot. In some homes, we collected two wipe samples to assess the variability in surface contamination between rooms. Our surface sampling followed the U.S. EPA protocol for lead dust wipe collection (Figure S2b).^39,40^ A detailed description of the sampling procedure is provided in the Supporting Information.

Metal concentrations in surface wipe samples were quantified using an inductively coupled plasma mass spectrometry (ICP-MS) instrument (Thermo iCAP-RQ, ASX-560 Autosampler) following acid digestion at the University of California, San Diego’s Environmental and Complex Analysis Laboratory (ECAL). Each wipe sample was treated with 10 mL of 1% HNO_3_, while the blank sample was processed identically using 20 mL of 1% HNO_3_. Samples were then filtered (0.2 μm PTFE) to reduce the total dissolved solids (TDS) content to <0.2%, which is preferable for ICP-MS analysis.^41^ Liquid samples were then diluted 100-fold in 1% HNO_3_ prior to analysis and introduced into the ICP-MS system. Calibration metal standards (Inorganic Ventures) were analyzed alongside the samples to quantify target metal concentrations. The acidified water samples were spiked with 50 μL (per 10 mL) ICP-MS 71D (Inorganic Ventures) prior to analysis. Each sample was measured in five replicate scans, and standard deviations of these replicates were used to estimate measurement uncertainty. The data were processed using Qtegra ICP-MS software (ThermoFisher Scientific).

### 2.4 Data Analysis

In this study, we processed TARTA’s measurements using a previously developed calibration model that links spectral intensity to the concentrations of target metals.^35^ The model was constructed using standard reference metal materials of known concentrations and has been validated and applied in prior field studies to quantify airborne metal particulates across diverse environmental settings.^36^ TARTA 2.0 can detect 16 metals (Al, As, Be, Cd, Co, Cr, Cu, Fe, Hg, Li, Mg, Mn, Ni, Pb, V, and Zn), with LODs ranging from 2.1 ng/m^3^ for Mn to 53.2 ng/m^3^ for As, based on a 30-min sampling resolution^38^ (Table S1).

Air exchange rates (ACH) were calculated using a mass balance model fitted to the observed indoor CO_2_ rise curves. Because at least one researcher remained indoors throughout sampling, CO_2_ concentrations increased (rather than decayed) over the 2–3-h sampling period, so the conventional CO_2_ decay and steady-state methods were not applicable (Figure S3). The mass balance approach was therefore used to estimate approximate ACH values for each household.^42^ Details of the model formulation and fitting procedure are provided in the Supporting Information, and the resulting ACH estimates are summarized in Table S5.

To evaluate whether differences in environmental conditions and postfire mitigation behaviors contributed to variations in indoor PM concentrations across homes, we conducted statistical comparisons using nine categorical predictor variables. These variables, derived from on-site observations and homeowner interviews, included window type, proximity to the fire zone, presence of persistent smoke odor, burned external structures, home occupancy status, HVAC use after the fire, closed windows or doors during the fire, indoor air purifier usage, and cleaning activities (Table S2). Each variable was categorized into two or three levels, representing distinct environmental or mitigation conditions. For example, proximity to the fire zone was categorized into three groups: backyard on fire, close (<100 m), and far (>100 m) from the fire zone or the nearest burned structure. Cleaning activity was divided into not cleaned, vacuum cleaning [non-high-efficiency particulate air (HEPA)], and professional cleaning, while window type was classified as single-pane, double-pane, or mixed. All other remaining variables were binary (yes or no). Because the raw PM concentration data were right-skewed and violated normality assumptions, values were log-transformed prior to analysis. Diagnostic plots^43^ (histograms and *Q*–*Q* plots, Figure S4) confirmed that the log-transformed data followed an approximately normal distribution, justifying the use of parametric tests. Two-sample *t* tests were used for binary variables, and one-way ANOVA (on log-transformed data) was used for variables with three levels to evaluate whether indoor PM concentrations differed significantly across categories.^44,45^

Variables that showed statistically significant variation in univariate tests (*p* < 0.05) were subsequently included in a multiple linear regression model to assess whether postfire indoor PM concentrations varied systematically across combinations of environmental and mitigation conditions in different homes. In this model, homes with the most adverse theoretical conditions—located closest to the fire, not cleaned, with no active air purifiers, and with doors or windows left open during the fire—were used as the reference category. Diagnostic plots (*Q*–*Q* plots) and Shapiro–Wilk^46^ tests confirmed the normality assumption of data for the regression model. To estimate outdoor PM_2.5_ and PM_10_ concentrations at each household, we applied a wind-adjusted inverse distance weighting (IDW) interpolation using data from the 11 PHOENIX PM monitoring stations.^47,48^ The basic IDW formulation is shown in eq 1:

1 $$\mathrm{outdoor}\;{\mathrm{PM}}_{i}=\frac{\overset{N}{\underset{j=1}{\sum }}\frac{{\mathrm{PM}}_{j}}{{{\mathrm{WCD}}_{ij}}^{2}}}{\overset{N}{\underset{j=1}{\sum }}\frac{1}{{{\mathrm{WCD}}_{ij}}^{2}}}$$

where outdoor PM_*i*_ is the estimated outdoor concentration at household *i*, PM_*j*_ represents the observed concentration at the *j*th PM station, and WCD_*ij*_ is the wind speed and direction-corrected distance weight factor between household *i* and station *j*.

## 3 Results

### 3.1 Indoor Air Pollutants

Figure 2 represents the distribution of indoor PM_2.5_, PM_10_, and BC concentrations across the 19 sampled homes. The U.S. EPA’s national ambient air quality standards (NAAQS) for PM_2.5_ and PM_10_ are shown as surrogate benchmarks for reference.^49^ Average PM_2.5_ concentrations (Figure 2a) in most homes remained below the EPA’s primary (i.e., health-based) annual standard of 9 μg/m^3^, and all measurements were below the primary 24-h standard of 35 μg/m^3^. The narrow interquartile ranges of PM concentrations indicate relatively small variability in indoor levels across the sampling period within each home. Notably, Home 4308 exceeded the 9 μg/m^3^ reference for most of the sampling period, while Homes 2307-1, 2307-2, and M3311 exhibited transient exceedances during the first 15 min of the sampling, likely due to a short-term spike caused by the resuspension of settled dust during instrument setup and the research team’s movement within the space. The mean PM_10_ concentrations (Figure 2b) across all homes were generally below the EPA’s 24-h standard of 150 μg/m^3^. Nonetheless, four homes (including Homes 2307-2, 4308, 3306, and M3311) showed maximum values exceeding this reference, with the first three also showing elevated PM_2.5_ levels. Of note, Homes 4308 and 3306 had undergone surface cleaning prior to sampling, while Homes 2307 and M3311 had fire reaching their backyards. These possible contributors to elevated PM concentrations are examined in greater detail in Section 4. The variation of BC concentrations across homes followed a pattern similar to that observed for PM_2.5_ (Figure 2c). Most of the BC concentrations measured in this study were below 500 ng/m^3^, falling well within the lower range reported in previous studies, where average BC levels in occupied residential environments generally remain under 1000 ng/m^3^.^50,51^ Interestingly, Home 1310 showed elevated BC levels (with an average above 1000 ng/m^3^) despite relatively low PM levels (see Section 4 for further discussion on potential contributing factors). Validated TVOC readings above the LOD of 10 ppb were observed in only three of the 19 households, with average measured concentrations of 42.5, 22.6, and 20.8 ppb in Homes 1307, 2307-1, and 2307-2, respectively. While there are no U.S. regulatory standards for indoor TVOC exposure, the World Health Organization (WHO) recommends an indoor exposure guideline of 125 ppb,^55^ a level below which all sampled homes fall. The relatively low TVOC levels observed also reflected the absence of major indoor emission sources during sampling. Potential confounding factors, such as emissions from cooking, cleaning products, and building materials, as well as indoor smoking, were minimal in our study because all homes were unoccupied during sampling, and no cleaning or other indoor activities occurred during measurement periods.^53^

**Figure 2:**
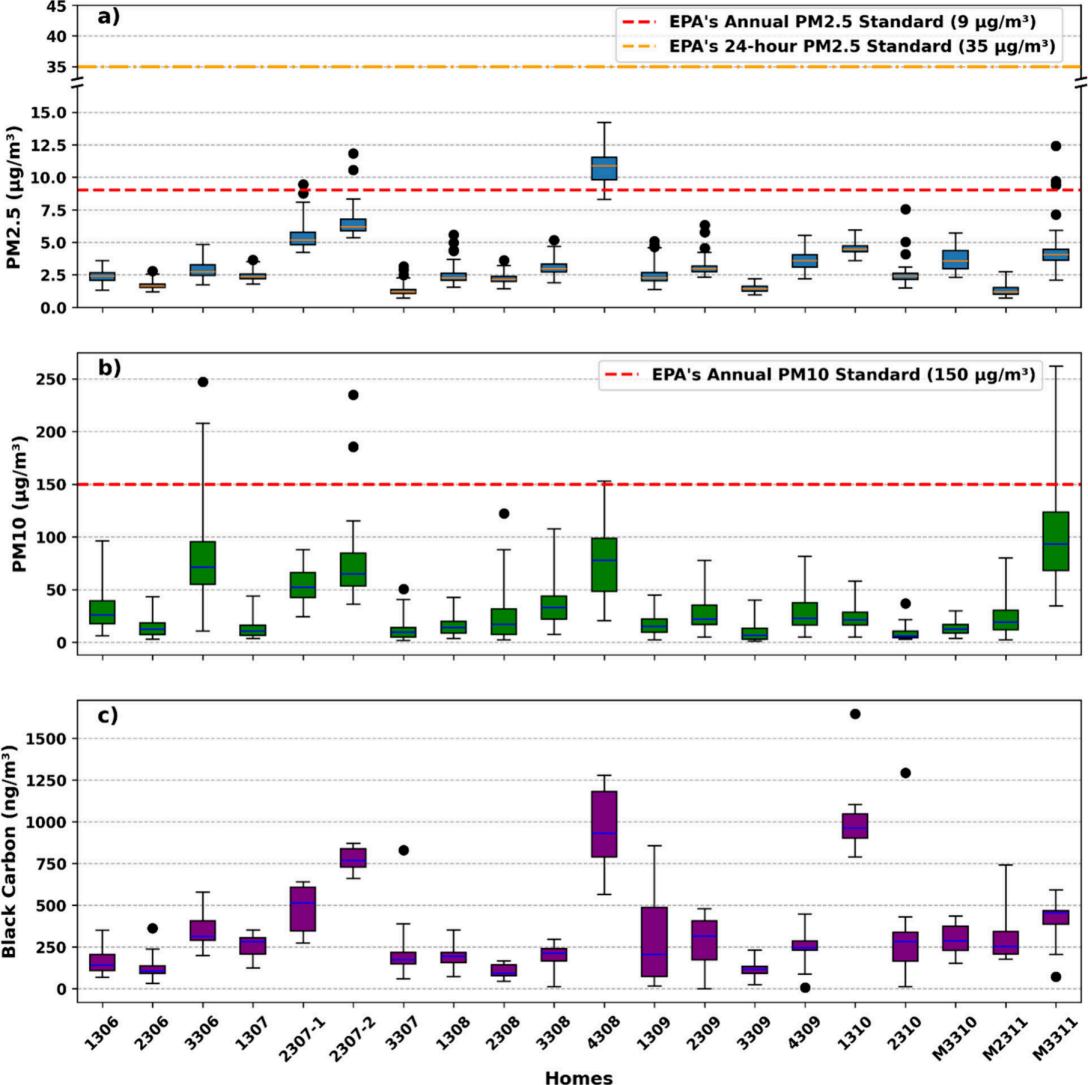
Variation of indoor concentrations of (a) PM_2.5_ (*y* axis is broken from 17 to 33), (b) PM_10_, and (c) BC across the sampled homes.

Indoor pollutant levels were found to be typically higher in occupied homes compared to unoccupied ones, where human activities (e.g., cooking, cleaning, smoking, ventilation practices, and infiltration from outdoor air) contribute directly to particle generation.^22,53,54^ However, in this study, of the four homes with PM concentrations exceeding the EPA standards, only Home 4308 was occupied at the time of sampling. While Homes 3307 (the benchmark home) and 2310 were also occupied, they comparatively exhibited lower concentrations, suggesting that occupancy alone may not account for elevated particulate levels in fire-affected homes; rather, other factors such as the effectiveness of postfire cleaning, ventilation practices, and the persistence of residual ash and soot may play a more significant role. This is particularly evident in our benchmark home, which differed from all other sampled residences by implementing the most protective measures (including professional cleaning, closed windows and doors during the fire, and consistent air purifier use) and consistently exhibited among the lowest concentrations of indoor PM (see Section 4 for further discussion).

Table 1 presents the average and standard deviation of elemental concentrations measured in indoor air across the 19 sampled homes. Although TARTA can detect up to 16 metal species (Table S1), only Al, Fe, and Mg were found above the instrument’s LODs in a majority of homes, with an average and standard deviation of 223.8 ± 153.9, 61.9 ± 52.3, and 305.1 ± 168.7 ng/m^3^ across all homes, respectively. The greatest Mg concentration was observed at Home 1306 (531.6 ng/m^3^), while Fe peaked at 183.8 ng/m^3^ at Home 3307 and Al peaked at 519.1 ng/m^3^ at Home M2311. Cu and Cr were detected in only a few homes: Cu was found in three homes, with a higher level of 5.9 ng/m^3^ at Home 3309, where Cr was also detected at an average of 5.8 ng/m^3^. The limited detection of other metals is not unexpected, as ambient elemental concentrations in the Eaton and Palisades regions were reported to reflect generally low urban levels. For example, SCAQMD’s analysis of filter samples using ICP-MS, which collected TSP similar to TARTA’s configuration, reported average ambient concentrations on March eighth and 11th of of 515 ng/m^3^ (Al), 447 ng/m^3^ (Fe), 242 ng/m^3^ (Mg), 12 ng/m^3^ (Cu), and 3 ng/m^3^ (Cr) at the Eaton sites and 1584 ng/m^3^ (Al), 1513 ng/m^3^ (Fe), 688 ng/m^3^ (Mg), 12 ng/m^3^ (Cu), and 5 ng/m^3^ (Cr) at the Palisades sites^9^ (a summary of the other elemental results from SCAQMD can be found in Table S3). Although few metals were detected by TARTA, this is a favorable outcome, as the measured concentrations of metals with established EPA reference inhalation concentrations (RfCs), including Be, Co, Cr­(VI), Hg, Mn, Ni, Pb, and V, were all below their respective RfC thresholds.^55^ However, residual ash deposited on indoor surfaces remains a concern (see Sections 3.3 and 4.2), though the low airborne metal concentrations suggest these particles are not being significantly resuspended into indoor air at the time of sampling.

**Table 1:** Airborne Elemental Concentrations Measured across 19 Homes (ng/m^3^)­^a^

	1306	2306	3306	1307	2307-1	2307-2	3307	1308	2308	3308
Al	<LOD	<LOD	392.2 ± 168.7	<LOD	<LOD	<LOD	<LOD	201.9 ± 116.2	<LOD	150.1 ± 126.7
Cr	<LOD	<LOD	<LOD	<LOD	<LOD	<LOD	<LOD	<LOD	<LOD	<LOD
Cu	<LOD	<LOD	3.2 ± 1.5	<LOD	<LOD	<LOD	<LOD	<LOD	<LOD	<LOD
Fe	18.9 ± 12.2	22.5 ± 11.3	96.1 ± 55.6	73.9 ± 24.6	37.1 ± 25.3	<LOD	183.8 ± 133.4	52.3 ± 28.1	<LOD	<LOD
Mg	531.6 ± 166	249.6 ± 43	524.8 ± 180.9	470.6 ± 113	220.9 ± 93.4	304.8 ± 16.8	176.5 ± 76.8	310.9 ± 208.8	121.9 ± 77.5	362.8 ± 88.9

	4308	1309	2309	3309	4309	1310	2310	M3310	M2311	M3311
Al	<LOD	121.9 ± 95.4	<LOD	206.1 ± 19.8	138.1 ± 108.7	<LOD	<LOD	<LOD	519.1 ± 27.2	61.3 ± 46.1
Cr	<LOD	<LOD	<LOD	5.8 ± 3.5	<LOD	<LOD	<LOD	<LOD	<LOD	<LOD
Cu	<LOD	<LOD	<LOD	5.9 ± 3.4	<LOD	<LOD	<LOD	<LOD	2.6 ± 1.2	<LOD
Fe	26.2 ± 5.5	<LOD	<LOD	<LOD	<LOD	<LOD	<LOD	<LOD	<LOD	48.2 ± 24.3
Mg	362.8 ± 88.9	<LOD	<LOD	<LOD	<LOD	<LOD	<LOD	<LOD	<LOD	<LOD

a Values represent mean ± standard deviation based on four 30-min indoor air samples collected at each home. The LODs of TARTA are 14.8 ng/m^3^ (Al), 4.2 ng/m^3^ (Cr), 2.4 ng/m^3^ (Cu), 11.8 ng/m^3^ (Fe), and 7.6 ng/m^3^ (Mg). “<LOD” in the table denotes concentrations below these LODs.

### 3.2 Indoor-to-Outdoor (I/O) Ratio of PM Concentrations

In this section, we use I/O PM ratios to assess how outdoor air quality affects indoor environments. An I/O ratio of >1 indicates indoor air is more polluted than outdoors, while an I/O < 1 suggests indoor air is cleaner than outdoors.^56^ Figure 3 presents I/O ratios for PM_2.5_ and PM_10_ across the sampled homes. These results are limited to homes in the Eaton fire-affected area, where both indoor and outdoor PM levels were measured using the same QuantAQ sensors. The average I/O ratio for PM_2.5_ was 1.08, with six homes exhibiting ratios of >1 (Homes 3306, 2307-1, 2307-2, 4308, 2309, and 1310). In contrast, PM_10_ ratios were higher, with an average of 2.99 and 12 out of 16 homes exhibiting I/O > 1. Three homes (3306, 2307-1, and 2307-2) had PM_10_ I/O ratios exceeding 5, with Home 3306 reaching a peak ratio of approximately 14.

**Figure 3:**
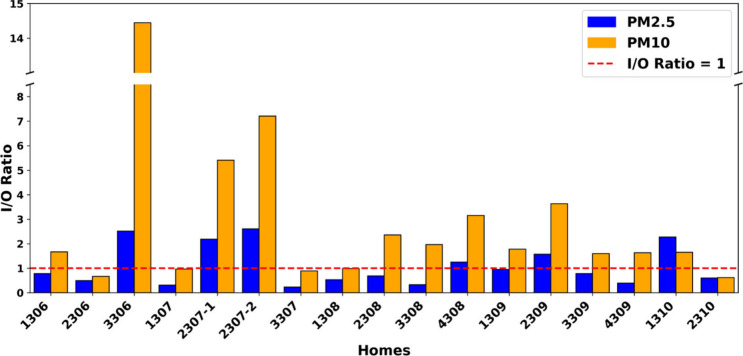
I/O ratios of PM_2.5_ and PM_10_ concentrations over all sampled homes in the Eaton Fire region. Note that the *y* axis is broken from 8.5 to 13.5.

Previous studies have reported that I/O ratios typically between 0.45 and 0.8 in residences during nonfire days, unless periodic indoor sources such as cooking or heating are present.^20,53,57,58^ In contrast, our study observed I/O ratios of >1 in many homes, particularly for PM_10_. Most of the homes in our study were unoccupied, had remained closed since the fire, and had not undergone any cleaning prior to sampling (with the exception of Homes 4308, 3307, and 2310). As such, the elevated I/O ratios are unlikely to result from occupant behavior or active indoor emissions during the sampling period but instead likely reflect the legacy of the fire smoke, as further explored in Section 4.1.

### 3.3 Surface Contaminants of Metals

Figure 4 presents the elemental concentrations of 14 metals in 30 surface wipe samples collected across all homes, including from carpets, tables, floors, countertops, and window sills. To ensure quantification accuracy, all reported metal concentrations were corrected by subtracting concentrations measured in field blanks, which were obtained as described in Section 2.3. Among all elements, K was the most abundant, dominating in 23 of the 30 samples, with an average concentration of 2235.4 ± 3054.2 μg/ft^2^ (Table S4). Mg, Al, and Fe were also prominent, averaging 1995.1 ± 3317.2, 1823.5 ± 2457.6, and 494.4 ± 694.3 μg/ft^2^, respectively. Their consistent presence across homes indicates widespread indoor contamination resulting from the inflation and deposition of local soil dust and burned vegetation residuals following the fires.^59,60^ Although we do not have measurements from unaffected (i.e., nonfire) homes for direct comparison, the two professionally cleaned homes in this study (3307 and 2310) provide a reasonable internal reference for postfire background conditions. Both showed markedly lower surface metal loadings than the study average, indicating that thorough cleaning substantially reduces residual contamination. In contrast, metal concentrations in most other homes were several times higher than these inferred baseline levels, underscoring the persistence of fire-related deposits on indoor surfaces.

**Figure 4:**
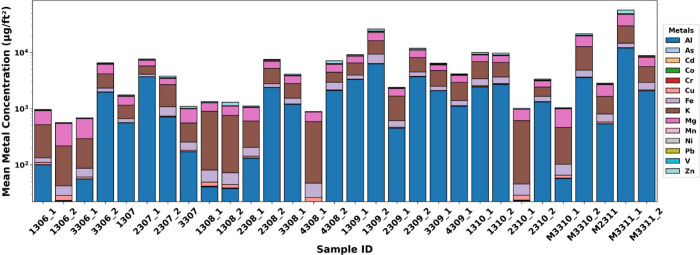
Concentration of 14 quantified metals from 30 surface wipe samples across all sampled households. Note that the *y* axis is on a log_10_ scale.

In addition to these crustal and biomass-related elements, Zn, Cu, and Mn were also detected, with average concentrations of 505.5 ± 1300.2, 26.6 ± 28.1, and 235.9 ± 450.1 μg/ft^2^ over all sampled homes, respectively. Zn and Cu are widely present in household infrastructure and vehicles, such as plumbing, wiring, roofing, and brake components, and are known to be released during combustion of these materials in WUI fires.^59^ Holder et al. (2023) found that Cu and Zn emission factors from vehicle combustion were over 60 and 400 times higher, respectively, than those from biomass burning.^12^ Mn, while naturally occurring in soils and vegetation, is also used as an additive in treated wood, steel, and metal coatings, which can volatilize at the high temperatures generated in structural fires.^59,61^

Pb also emerged as a key anthropogenic contaminant in our surface wipe samples. As shown in Figures 5 (floors and surface samples) and S5 (window sill samples), Pb concentrations exceeded the EPA’s dust lead clearance levels of 5 μg/ft^2^ for floors and surfaces and 40 μg/ft^2^ for window sills in multiple households, with values at least four times greater than the standard. Given that most homes in the fire-affected regions were likely built before 1978 (the year that the U.S. banned the residential use of Pb-based paints), as evidenced by all 19 sampled homes in this study, the probable use of such paints in these buildings could be a significant contributor to the elevated Pb levels detected on indoor surfaces.^62–64^ Additionally, the combustion of electric vehicles and internal combustion engine vehicles can also serve as a contributor to the observed Pb contamination.^65^

**Figure 5:**
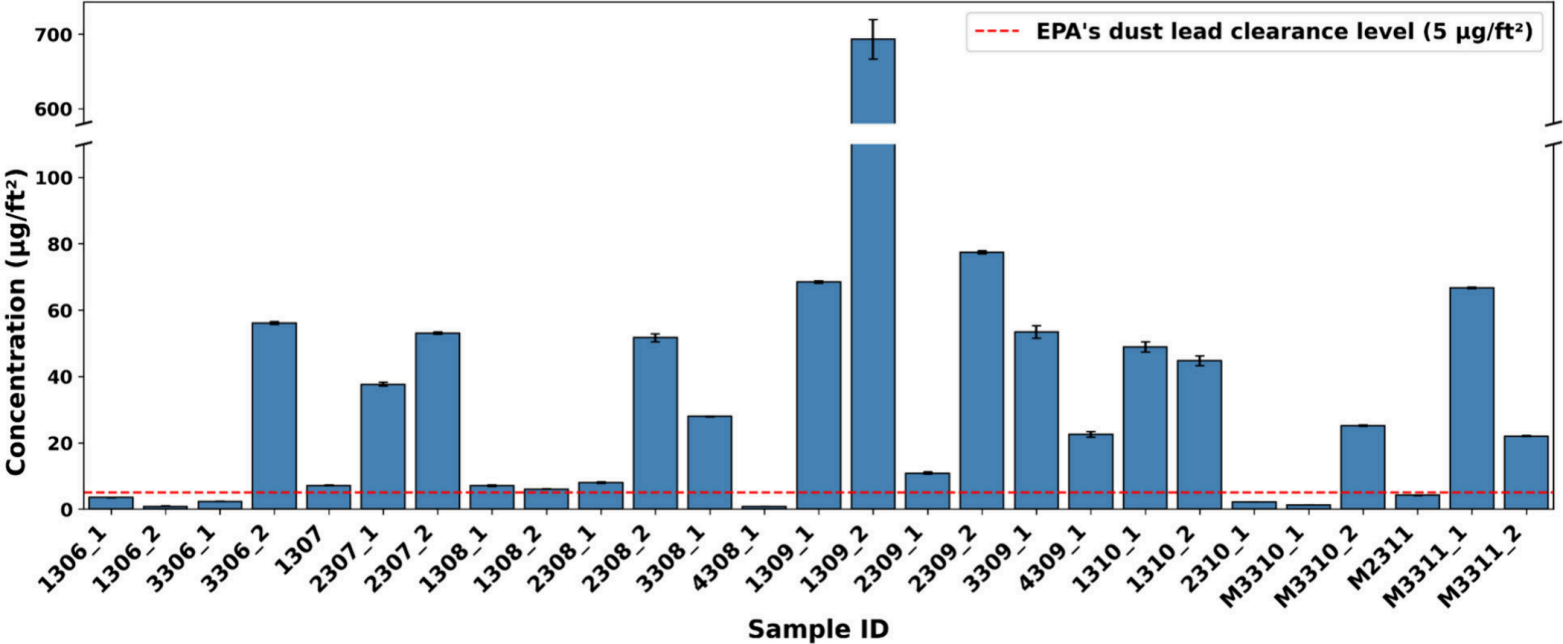
Pb concentrations in wipe samples taken from floors and surfaces across sampled homes. Note that the *y* axis is broken from 110 to 580. A similar figure of Pb concentration taken from window sill samples can be found in Figure S5.

Previous studies outside fire-affected regions reported average surface loadings of approximately 0.7, 12.2, 4.5, and 1.2 μg/ft^2^ for Mn, Zn, Cu, and Pb, respectively. In comparison, our measurements from households affected by the Eaton and Palisades Fires showed consistently higher metal loadings than these typical background levels observed in the absence of WUI fires.^66,67^

Comparisons across surface types further reveal distinct patterns of metal deposition, reflecting how material characteristics influence indoor contaminant distribution. Window sills frequently exhibited higher metal concentrations than adjacent surfaces, as illustrated by Sample 4308_2 versus 4308_1 and 2310_2 versus 2310_1, likely due to direct deposition from outdoor air infiltration (Figures 5 and S5). Similarly, entrance floor surfaces (e.g., Samples 2309_2, 1310_1, and M3310_1) exhibited elevated metal concentrations, likely due to particulate intrusion through door gaps or door opening during the fire. In contrast, wardrobes and closets (e.g., Samples 2310_2 and M3310_1) had lower concentrations, likely due to their enclosed and protected positions. Carpeted surfaces (e.g., Samples 1306_1, 3306_1, and 4308_1) consistently exhibited low metal concentrations compared to other indoor surfaces. This may be attributed to their fibrous and porous structure, which both limits surface deposition and reduces recovery efficiency during wipe sampling. Prior research findings note that while carpets can act as long-term particle reservoirs, they are less effective for surface sampling compared to smooth, hard materials.^68,69^

## 4 Discussion

### 4.1 Possible Causes of I/O Ratios of >1 2 Months after the Fire

To understand the drivers of PM I/O ratios of >1 in many of our sampled homes after 2 months of the fire (Figure 3), it is important to assess whether they are due to changes in indoor concentrations, outdoor concentrations, or both. In particular, an I/O ratio of >1 may reflect low outdoor PM levels rather than high indoor levels during the sampling period. To investigate this, Table S5 presents the average and standard deviation of PM_2.5_ and PM_10_ concentrations measured at both indoor and outdoor locations across all homes. The variation in outdoor PM concentrations was relatively small during our sampling periods, with a total mean and standard deviation of 4.31 ± 2.71 μg/m^3^ for PM_2.5_ and 13.12 ± 5.11 μg/m^3^ for PM_10_. This consistency of outdoor PM levels across homes suggests that the higher I/O ratios observed in some homes were primarily driven by elevated indoor PM concentrations there, rather than by fluctuations in outdoor conditions during the sampling period.

As mentioned in Section 3.2, most homes were unoccupied and had no active indoor sources, suggesting that the elevated indoor PM concentrations were related to fire debris. Below, we propose three mechanisms to explain these elevated I/O ratios: investigator-effect resuspension, low-ACH trapping, and high-ACH disturbance in heavily contaminated areas.

The first mechanism is resuspension of settled fire residues through human activity.^22^ Walked-on floors and lightly disturbed surfaces can reaerosolize particles even under minimal movement (e.g., the movement and equipment setup of our sampling team), which we call the “investigator effect” (included as an explanatory variable in the regression model described in Section 4.2). This investigator effect was evident in 7 out of 20 samples (Homes 3306, 2307, 2308, 3308, 4308, 3309, and 1310), where PM concentrations peaked during instrument setup and decayed once indoor activity subsided (Table S5 and Figure S6). Nevertheless, these homes still maintained overall I/O > 1 throughout the sampling period, indicating that resuspended particles remained airborne over the 2–3-h sampling duration.

The second mechanism involves low air exchange rates that trap resuspended particles indoors.^70^ After the fire, and even during our sampling, some households kept doors and windows closed to minimize the intrusion of postfire smoke and residual outdoor particulates. In Homes 3306, 2308, 3308, and 4308, the estimated air change rates were on average 0.34 h^–1^, which is lower than typical residential benchmarks of 0.5 to 1 h^–1^ for naturally ventilated homes.^42^ In these homes, limited ventilation suppresses dilution and allows resuspended particles to accumulate rather than disperse. The combination of low-ACH trapping with the investigator effect described above likely accounts for the persistently high I/O ratios in these homes.

In contrast to the second mechanism, the third involves high-ACH turbulence-driven resuspension in heavily contaminated entry zones. Several homes with the highest ventilation rates (Homes 2307, 1309, 2309, 3309, 4309, and 1310) also exhibited I/O > 1, even though elevated air exchange would normally be expected to reduce indoor PM. We hypothesize that high ventilation elevates indoor PM by generating turbulent airflows that re-entrain ash residues from heavily contaminated surface areas, such as entry floors, windows, and vents (Figure S7). Prior chamber studies have shown that even modest air velocities (∼0.3 m/s) can re-entrain coarse particles from ducts or floors.^71,72^ In such cases, high ventilation may accelerate decay after the initial investigator-caused spike yet still elevate time-averaged PM concentrations through repeated localized resuspension.

It is important to note that the effect of ACH on indoor particle levels depends strongly on the presence of residual fire debris. In homes where ash and soot had been thoroughly removed, the two professionally cleaned residences consistently exhibited I/O < 1, regardless of ventilation rate (3307 with ACH < 0.5 h^–1^ and 2310 with ACH > 1 h^–1^). This finding highlights that thorough cleaning through the removal of residual surface debris plays a more decisive role than ventilation rate alone in improving postfire indoor air quality.

The pronounced elevation of PM_10_ I/O ratios compared to PM_2.5_ arises primarily from size-dependent resuspension dynamics. PM_10_ particles, due to their greater mass and weaker adhesive forces, detach far more easily than fine particles under similar mechanical forces. In a controlled chamber study, PM_10_ resuspension rates were found to be an order of magnitude higher than PM_2.5_ [∼0.25 h^–1^ (PM_10_) vs ∼0.02 h^–1^ (PM_2.5_)] during identical walking simulations.^73^

### 4.2 Key Factors Driving PM Variation across Homes

Our individual parametric tests suggested six variables (including proximity to the fire zone, presence of persistent smoke odor, burned external structures, closed windows or doors during the fire, indoor air purifier usage, and cleaning activity) were each associated with significant variations in PM concentrations between their respective categories across the sampled homes (*p* < 0.05) (Table S2). However, in the multiple linear regression model, we excluded burned external structures (due to overlapping with fire proximity) and persistent smoke odor (due to its subjective nature) as independent variables. To account for potential particle resuspension caused by the research team’s movement during instrument setup, we included an “investigator effect” variable as an explanatory factor in the regression model. The final model was built upon the remaining four variables, which explain 78% and 87% of the variance in indoor PM_10_ and PM_2.5_ concentrations, respectively, across all sampled homes (Table 2).

**Table 2:** Multiple Linear Regression Results on Average Indoor PM Concentrations

variables	reference condition	PM_10_ regression coefficients^a^	PM_10_ *p* value^b^	PM_2.5_ regression coefficients	PM_2.5_ *p* value
model intercept		66.6	***	4.9	***
distance from fire zone of <100 m	backyard on fire	–32.5	***	–1.1	***
distance from fire zone of >100 m		–34.3	***	–0.7	0.25
professional cleaning	not cleaned	–11.7	0.46	–0.3	0.72
vacuum cleaning (non-HEPA)		26.3	***	6.3	***
active air purifiers	w/o air purifier	–33.3	***	–1.1	0.14
windows or doors were fully closed during the fire	windows or doors were opened during the fire	–11.8	0.33	–1.5	***
investigator effect	no initial peak in the PM levels	15.2	0.14	0.5	0.36
		number of samples*s* = 20	*R* ^2^ = 0.78	number of samples*s* = 20	*R* ^2^ = 0.87

a Regression coefficients represent the change in indoor PM (μg/m^3^) across homes associated with each condition, relative to the reference category. A negative coefficient indicates a reduction in PM compared to the reference.

b Statistically significant *p* values (i.e., *p* < 0.05) are indicated as ***.

Proximity to the fire zone emerged as one of the major predictors of indoor PM_10_ levels. Compared to homes with their backyard directly on fire, homes located less than 100 m away from the fire had an average reduction of 32.5 μg/m^3^ (*p* < 0.05), and those more than 100 m away showed a reduction of 34.3 μg/m^3^ (*p* < 0.05). Although both distance categories were associated with significantly lower PM_10_ levels relative to the reference group, the comparable reductions in PM_10_ levels suggest that additional site-specific factors can influence the difference of postfire PM levels in different homes, such as wind direction during the fire, the orientation of windows and doors relative to prevailing winds, proximity to major roads, and household infiltration characteristics.^53^

The use of air purifiers after the fire was associated with a substantial reduction of 33.3 μg/m^3^ in PM_10_ (*p* < 0.05) compared to homes without them. This finding reinforces the effectiveness of active filtration systems in mitigating PM exposure in fire-affected homes, consistent with recent studies that have demonstrated the efficacy of HEPA purifiers in reducing indoor PM levels during and after high-pollution events, including wildfire episodes and dust storms.^74–76^

The impact of cleaning activities on indoor PM_10_ concentrations varied substantially between homes. While professional cleaning was associated with a reduction of 11.7 μg/m^3^ relative to uncleaned homes, the reducing effect was not significant (*p* = 0.46). In contrast, non-HEPA vacuum cleaning was associated with a significant increase of 26.3 μg/m^3^ in indoor PM_10_ concentration (*p* < 0.05), suggesting that standard household vacuums may resuspend ash and soot particles, worsening the IAQ. More specifically, Homes 3307 and 2310 underwent deep cleaning. While there is no specific definition for deep cleaning, it can be broadly described as cleaning methods that include the use of wet cloths, mops, and HEPA-filter vacuums, methods known to limit particle resuspension.^77,78^ These homes were associated not only with lower indoor PM_10_ levels but also with surface lead dust concentrations below the EPA standards, as mentioned in Section 3.3. In contrast, Home 4308 was cleaned using a common household vacuum without a HEPA filter, along with wet cloth wiping. Previous studies have shown that such vacuums are ineffective at capturing fine particles and may recirculate contaminants into the indoor air.^79,80^ Additionally, residents at Home 4308 reported using a leaf blower to clear ash and soot from the front lawn, which could further contribute to indoor particle loading and surface lead dust by transporting outdoor contaminants indoors.

Keeping windows and doors fully closed during the fire resulted in a modest reduction of 11.8 μg/m^3^ in PM_10_ compared to homes with openings, though this effect is not a significant predictor of PM_10_ (*p* = 0.33). The limited association of this variable with different PM_10_ levels across homes may be explained by uncontrolled variability in building leakage and the timing of window closure after the fire started, both of which require further investigation that was not systematically documented in this study. It is also important to note that during the fires, strong Santa Ana winds with gusts reaching up to 100 mph were blowing through the Eaton and Palisades Fires regions. Although homeowners reported closing windows and doors prior to evacuation, these winds likely forced windows and doors open in some homes and transported ash and smoke indoors through these openings, as well as ventilation ducts, air gaps, and structural cracks. Nonetheless, previous research supports our findings that maintaining a well-sealed building envelope (by keeping windows and doors closed and sealing cracks) can significantly reduce indoor PM levels during wildfire events, especially compared to homes with higher infiltration rates.^13^

The investigator effect was associated with an average increase of 15.2 μg/m^3^ in PM_10_ compared to homes without the initial peak–decay pattern. As mentioned before, this increase is mainly due to the instrument setup and sampling activities during the first 15 min after entry. However, the investigator effect was not a statistically significant predictor of PM_10_ levels (*p* = 0.14).

The PM_2.5_ regression model shown in Table 2, which included the same four predictor variables, exhibited coefficient signs consistent with those in the PM_10_ model (i.e., PM_2.5_ levels tended to be lower in homes equipped with air purifiers, located farther from the fire, and with windows and doors closed during the fire; and higher in homes using non-HEPA vacuums or situated closer to the fire). However, only the categories of <100 m distance, non-HEPA vacuuming, and closed windows and doors during the fire were statistically significant in explaining the variation in average PM_2.5_ concentrations across homes.

### 4.3 Hazardous Air Pollutants in Surface Wipes

Initial air quality reports from the early days of the Eaton and Palisades fires documented acute spikes in airborne Pb concentrations. At the Pico Rivera ASCENT station (roughly 30 miles from the Eaton fire region), for instance, Pb levels exceeded 100 times the typical background average on January ninth, indicating substantial short-term emissions from the combustion of manmade materials.^7^ However, these spikes subsided within approximately 48 h of fire onset. Subsequent continuous ambient air monitoring conducted by the SCAQMD beginning in February showed that concentrations of the seven metals classified as hazardous air pollutants (HAPs) by the U.S. EPA (including As, Cd, Cr, Co, Mn, Ni, and Pb) had returned to typical background levels at the Los Angeles Basin^9^ (Figure S8 and Table S3). Similarly, our indoor air measurements, conducted approximately 2 months after the fires, detected no elevated concentrations of these metals; most were below TARTA’s LODs or comparable to concurrent outdoor levels (see Section 3.1).

However, the absence of elevated airborne metal concentrations does not imply the absence of indoor residual contamination and exposure risks. Our surface wipe samples revealed that all seven HAP metals were detected in multiple homes (Figure S8). These metals likely infiltrated when ambient concentrations were high and settled indoors along with ash and soot during or shortly after the fire. Among the HAP metals detected in surface wipe samples, Pb and Mn were the most abundant metals and exhibited elevated levels in a subset of homes (including 16 out of 30 wipe samples) compared to the benchmark home (Home 3307). This pattern aligns with SCAQMD ambient air measurements during the postfire monitoring period, where Pb, Mn, and Cr were the most elevated metals among the HAPs (Figure S8). A notable example is Sample 1309_2, collected from a garage floor, which shows one of the highest concentrations of all seven HAP metals, particularly Pb and Mn, highlighting how semiexposed or outdoor areas can serve as metal accumulation zones.

These findings suggest that even after airborne levels of HAP metals have declined, indoor surfaces may act as persistent reservoirs, capable of resuspending metal particles into the air through certain environmental disturbances, such as walking, sweeping, and vacuuming. This risk is especially pronounced in homes that have not undergone thorough postfire remediation and poses particular concerns for vulnerable populations, including elders and children, who are more susceptible to the harm through inhalation and dermal exposure. In addition to indoor sources, postfire soil and surrounding ground surfaces represent another potential source of re-exposure. The accumulated ash and soot in these areas can be remobilized by strong winds, debris removal, or the use of leaf blowers, contributing not only to outdoor air contamination but also to subsequent indoor exposure through infiltration.

## 5 Summary and Implications

This study presents a comprehensive evaluation of indoor air quality and surface contamination in 19 homes affected by the Eaton and Palisades fires, approximately 2 months after fire events. Indoor PM concentrations were generally below U.S. EPA ambient air standards across homes, with overall average PM_2.5_ and PM_10_ concentrations of 3.45 and 31.66 μg/m^3^, respectively. BC levels were found to be below typical values reported for occupied homes, reflecting the unoccupied status of most sampled homes. Notably, I/O ratios of >1 were observed in many homes, particularly for PM_10_, revealing a previously unreported postfire pattern in which both low and high ventilation rates can sustain elevated indoor PM concentrations. Specifically, low ACH can limit dilution and trap resuspended fire residues within the indoor environment, while high ACH can induce localized turbulence that mobilizes and re-entrains ash and soot from highly contaminated indoor surfaces. Regression analysis of key environmental and behavioral factors revealed that proximity to the fire zone, closed windows or doors during the fire, indoor air purifier usage, and cleaning activity significantly influenced indoor PM levels, explaining 78% of PM_10_ and 87% of PM_2.5_ variations across all homes. Although indoor airborne metal concentrations during the study period were within typical background ranges, surface wipe samples revealed widespread, and in some cases elevated, levels of metals on indoor surfaces. Detected metals appear to originate from different WUI fire sources: K, Mg, and Fe were consistent with vegetation combustion, while Pb, Zn, Cu, and Cr likely originated from infrastructure materials. The presence of HAP metals, including Pb and Mn, on indoor floors and surfaces 2 months postfire, exhibits that these metals can persist on surfaces and act as potential reservoirs for re-exposure, especially through resuspension, posing long-term health risks even when air quality appears improved. Of note, surface metal concentrations varied by location, with window sills and entry floors showing the highest levels, and carpeted or enclosed interior areas the lowest.

This study is subject to several limitations, including the relatively small sample size, the limited number of professionally cleaned homes as of sampling period, and the absence of sampling at homes located much farther from the fires, which may have constrained our interpretation. Nonetheless, consistent with previous studies, our results emphasize the importance of comprehensive postfire cleaning and remediation practices, as homes that underwent wet wiping and HEPA vacuuming showed reduced indoor PM and metal levels. Furthermore, given the rapid deployment of this study, we were unable to include the necessary equipment for speciated organic analyses of indoor air and dust during the first sampling round.

As we move into the second phase of postfire recovery, and with ambient air quality expected to continue improving, future work will address community concerns regarding elevated indoor air pollution during hot weather due to higher ventilation rates and increased off-gassing from fire-affected surfaces and furniture. Additionally, with more residents returning and a greater number of homes having undergone professional cleaning, upcoming efforts will focus on evaluating postremediation indoor air quality and surface contamination. These assessments will be compared to baseline conditions reported in this paper and will also be used to evaluate the effectiveness of different cleaning strategies across households.

## Supplementary Material



## Notes

Additional sampling and ACH calculation methods, questionnaires, statistical results, and supplementary tables and figures (PDF)
