# Supplementary material for: Post-Wildfire Indoor Pollution in WUI Areas following the 2025 Los Angeles Fires. Part I. Establishing Baseline Contaminant Levels Prior to Home Reoccupation
Source: ACS EST Air. 2025 Dec 19;3(1):142–55. doi: 10.1021/acsestair.5c00281 (PMC12797226; doi:10.1021/acsestair.5c00281)
Supplement: Supplementary file 1 [file ea5c00281_si_001.pdf]

Supplementary Information -

Post-Wildfire Indoor Pollution in WUI Areas Following the 2025 Los Angeles Fires — Part I: Establishing Baseline Contaminant Levels Prior to Home Reoccupation

Ehsan Goftari†‡, Jose Rivera Carias†, London Fulford†, and Hanyang Li†*

† *Department of Civil, Construction, and Environmental Engineering, San Diego State University, San Diego, California 92182, United States*

‡ *Department of Mechanical and Aerospace Engineering, University of California San Diego, La Jolla, California 92037, United States*

* Email: hli6@sdsu.edu

This Word document includes:

- Supplementary Text (Pages 2-4)
  - Questionnaire to recruit volunteers
  - Sampling procedure of the dust wipes
  - ACH Calculation
- Tables S1 to S5 (Pages 5-12)
- Figures S1 to S8 (Pages 13-16)

SUPPLEMENTARY TEXT

- **Questionnaire to recruit volunteers**

Post-fire indoor air monitoring

Coordinating research activities for the research team from San Diego State University (Hanyang Li, hli6@sdsu.edu). The team will spend approximately four hours at your home, using battery-powered, portable air quality monitors to measure particulate matter (PM), volatile organic compounds (VOCs), and toxic metals inside and outside your home. They will also test air purifiers to evaluate their effectiveness in reducing pollution. Their presence will cause minimal disruption, and you can continue your normal activities while monitoring takes place. Your participation will help provide valuable data to improve post-fire recovery strategies and protect community health. The information provided below will be shared with the research team. Monitoring is expected to start as early as 3/3/25.

* Indicates required question

1. **Your Name** *
2. **Phone Number** *
3. **Email** *
4. **Address of home in/near the Eaton Fire impacted area** *
5. **Can you smell wood smoke or other burning odors inside your home?** *
   - Yes
   - No
   - Other:
6. **What is your best estimate of your home's fire damage status?** *
   - Destroyed (>50%)
   - Major (26% – 50%)
   - Minor (10% – 25%)
   - Affected (1% – 9%).
   - No structural damage, but impacted by smoke and ash
   - Other:
7. **When did you first return to your fire-affected home?** *
   - Before evacuation orders were lifted
   - Within a day after the evacuation orders were lifted
   - Within a week after the evacuation orders were lifted
   - More than a week after the evacuation orders were lifted
8. **Do you currently have power in your home?** *
   - Yes, electricity was not damaged during the fire
   - Yes, grid electricity has been restored after the fire
   - Yes, we rely on a generator provided by the county
   - No, power is still unavailable
   - Other:
9. **Have you already cleaned the ash inside your home?** *
   - Yes, by wiping surfaces
   - Yes, using air purifiers
   - Yes, by vacuuming or washing
   - Not yet
   - Other:

10. Do you have plans to do any reconstruction or repairs for structural damage? *

- Yes
- No
- Other:

11. I have the authority and give consent for researchers to access and take/remove samples of soil and ash from my property at the address given above. (If no, no samples will be collected.) *

- Yes
- No

12. Is there anything else you would like us to know about your home's condition or your concerns about indoor air quality?

Note: All study procedures involving human participants were conducted in accordance with the ethical standards of the Institutional Review Board of San Diego State University.

- **Sampling procedure of the dust wipes**

Our surface sampling followed the U.S. EPA protocol for lead dust wipe collection, using a 3D-printed template with a 10 × 10 cm^2 opening to define the sampling area. For each sample, the template was secured to the target surface using painter's tape. To prevent cross-contamination, a new pair of gloves and a Kimtech delicate task wiper, pre-wetted with deionized water in a petri dish, were used for each sample. To collect the dust sample, we placed the wipe at the corner of the template and started a sideways (i.e., horizontal) motion. Then, we folded the wipe and used the clean side to wipe the surface in a forward and backward (i.e., vertical) motion, starting from the same corner. Finally, we folded the wipe again and used another clean side to clean the interior edges of the sampling area. Once the sample collection was done, the wipe was folded inward with the sample side enclosed, placed in a sanitized centrifuge tube, and sealed. All the tubes were labeled with date, house ID, location of sample collection, and stored in a transport container. To assess background contamination, we collected field blanks at the beginning and end of each sampling day. These wipes were handled identically to sample wipes but were not used to contact any surfaces. All samples were stored in a freezer upon return to the laboratory prior to analysis.

- **ACH calculation**

We employed a mass balance model as shown in Equation 1 to describe the temporal evolution of CO_2 concentration:

$$\mathrm{V}\frac{\mathrm{dC}}{\mathrm{dt}} = \mathrm{E} + \mathrm{QC_R} - \mathrm{QC_{in}} \quad (1)$$

where V is the volume of the house (m^3), E is the indoor CO_2 emission rate ($mg.h^{-1}$), Q is the flow rate of outdoor air ($m^3.h^{-1}$), CR is the outdoor CO_2 concentration (ppm), and Cin is the indoor CO_2 concentration (ppm). Dividing both sides by V and rearranging yields:

$$\frac{dC}{dt} = \frac{E}{V} + n\mathrm{C_R} - n\mathrm{C_{in}} \quad (2)$$

where n is the ACH (=Q/V, hr^{-1}).

Using the initial condition ($C(0) = C_0$), the analytical solution to this differential equation is:

$$C(t) = (C_R + \frac{E}{Vn}) + (C_0 - C_R - \frac{E}{Vn})e^{-nt} \quad (3)$$

Under a steady-state condition ($\frac{dC}{dt} = 0$), the equilibrium concentration is $C_{ss} = C_R + \frac{E}{Vn}$. Substituting this into Equation 3 gives:

$$C(t) = C_{ss} + (C_0 - C_{ss})e^{-nt} \quad (4)$$

The model was fitted to the observed indoor CO_2 rise curves (an example shown in Figure S3) to estimate C_{ss} and ACH for each household. The fitting procedure began by assuming an initial C_{ss}, calculated as the mean of the last 10% of recorded CO_2 values at each home, which represented the period closest to steady state under our measurement conditions. Using this estimate, an initial ACH value was derived, and the residuals were obtained by subtracting the modeled C(t) from the measured concentrations. Both C_{ss} and ACH were then iteratively optimized to minimize the sum of squared residuals, thereby achieving the best fit to the measured data.

SUPPLEMENTARY TABLES

Table S1. TARTA 2.0 LODs based on 30-minute sampling duration. All the units are in ng/m³.

Metal	Al	As	Be	Cd	Co	Cr	Cu	Fe	Hg	Li	Mg	Mn	Ni	Pb	V	Zn
LOD (1σ)	14.8	53.4	10.4	20	3.4	4.2	2.4	11.8	16.8	4	7.6	2.1	20.8	14	4.1	2.8

Table S2. Categorical variables and t-test results for indoor PM concentration differences across homes

Home ID	Type of windows	Burned external properties	Proximity to the fire	Reoccupation	Persistent smoke odor	Air purifier usage	HVAC use after the fire	Closed windows and doors during the fire	Cleaning activity	Floor Area (m^2)
**3311**	Single-pane	External structure	Backyard on fire	No	Yes	No	No	No	Not cleaned	195
**1306**	Double-pane	No	<100m	No	Yes	No	No	Yes	Not cleaned	114
**2306**	Double-pane	No	>100m	No	No	No	No	Yes	Not cleaned	109
**1307**	Single-pane	No	>100m	No	Yes	No	No	Yes	Not cleaned	169
**2307**	Single-pane	External structure	Backyard on fire	No	Yes	No	No	No	Not cleaned	285

Home ID	Type of windows	Burned external properties	Proximity to the fire	Reoccupation	Persistent smoke odor	Air purifier usage	HVAC use after the fire	Closed windows and doors during the fire	Cleaning activity	Floor Area (m^2)
**3307**	Double-pane	No	>100m	Yes	No	Yes	Yes	Yes	Deep cleaning (Early February)	160
**1308**	Mixed	No	<100m	No	Yes	No	No	Yes	Not cleaned	132
**2308**	Single-pane	No	<100m	No	Yes	No	Yes	Yes	Not cleaned	187
**3308**	Mixed	No	>100m	No	Yes	No	No	Yes	Not cleaned	107
**4308**	Double-pane	No	<100m	Yes	No	No	Yes	No	Vacuum cleaning (Late January)	215
**1309**	Single-pane	No	<100m	No	Yes	No	No	Yes	Not cleaned	142
**2309**	Single-pane	No	<100m	No	Yes	No	No	No	Not cleaned	133
**3309**	Single-pane	No	>100m	No	No	Yes	No	Yes	Not cleaned	84
**4309**	Mixed	No	<100m	No	Yes	No	No	No	Not cleaned	228
**1310**	Mixed	No	>100m	No	No	No	No	Yes	Not cleaned	388

Home ID	Type of windows	Burned external properties	Proximity to the fire	Reoccupation	Persistent smoke odor	Air purifier usage	HVAC use after the fire	Closed windows and doors during the fire	Cleaning activity	Floor Area (m^2)
**2310**	Single-pane	No	>100m	Yes	No	Yes	No	Yes	Deep cleaning (Late January)	149
**3310**	Double-pane	External structure	Backyard on fire	No	Yes	Yes	No	Yes	Not cleaned	465
**2311**	Mixed	No	Backyard on fire	No	No	Yes	Yes	Yes	Not cleaned	306
**3306**	Mixed	External structure	Backyard on fire	No	Yes	No	No	Yes	Not cleaned	251
**PM2.5 p-value[1]**	***	***	***	***	***	***	***	***	***	---
**PM10 p-value[1]**	0.35	***	***	0.92	***	***	0.91	***	***	---

[1] Statistically significant p-values (i.e., $p < 0.05$) are indicated as ***.

Table S3. Elemental concentration detected at the stationary air monitoring stations of South Coast AQMD. Note: Only metals detectable by the TARTA are included.

Legend Name	Sample Date	Al	As	Be	Cd	Co	Cr	Cu	Fe	Li	Mg	Mn	Ni	Pb	V	Zn
**Eaton Site #3**	**3/8/2025**	351.59	0.21	ND	ND	0.16	3.04	6.32	385.49	ND	244.4	6.53	1.08	2.45	0.69	19.67
**Eaton Site #2**	**3/8/2025**	486.52	0.28	ND	ND	0.24	2.42	15.45	477.11	0.67	260.7	8.64	1.18	3.88	0.94	24.73
**Eaton Site #1**	**3/8/2025**	568.02	0.2	ND	ND	0.2	3.8	26.57	375.02	ND	312.37	7.78	2.11	1.56	0.72	28.61
**Eaton Site #3**	**3/11/2025**	468	0.16	ND	ND	0.19	2.9	5.1	410.41	ND	147.1	8.14	0.98	2.26	0.83	19.7
**Eaton Site #2**	**3/11/2025**	700.72	0.26	ND	ND	0.27	3.83	6.57	586.01	ND	245.23	11.56	1.49	3.58	1.17	28.21
**Palisades Site #1**	**3/8/2025**	2331.4	0.62	0.09	0.15	1.01	5.63	11.47	2102	1.59	980.07	39.05	3.1	3.47	4.31	64.16
**Palisades Site #2**	**3/8/2025**	906.39	0.39	ND	0.09	0.43	4.09	12.02	819.89	0.73	567.24	15.43	2.21	2.51	1.62	33.33
**Palisades Site #1**	**3/11/2025**	1514.1	0.51	ND	ND	0.63	5.89	13.72	1616.2	1.15	516.82	26.29	2.9	3.32	2.92	45.86
**Average (Eaton)**		515 ± 129	0.2 ± 0.05	ND	ND	0.2 ± 0.04	3.2 ± 0.6	12 ± 9	447 ± 87	ND	242 ± 60	8.5 ± 1.9	1.4 ± 0.5	2.7 ± 1	0.9 ± 0.2	24 ± 4
**Average (Palisades)**		1584 ± 715	0.5 ± 0.1	ND	0.12 ± 0.04	0.7 ± 0.3	5.2 ± 1	12 ± 1	1513 ± 647	1.2 ± 0.4	688 ± 254	27 ± 12	2.7 ± 0.5	3 ± 0.5	3 ± 1	48 ± 16

Table S4. Metal concentrations in 30 wipe samples across sampled homes. Values represent mean ± standard deviation based on five repeated analyses of each sample.

Sample ID	Al	As	Cd	Co	Cr	Cu	Fe	K	Mg	Mn	Ni	Pb	V	Zn
1306_1	99.28 ± 7.09	0.87 ± 0.07	0.16 ± 0.03	0.12 ± 0.02	0.17 ± 0.01	9.96 ± 0.06	22.42 ± 2.28	385.73 ± 11.03	400.17 ± 14.40	9.80 ± 0.29	1.66 ± 0.13	3.46 ± 0.04	0.66 ± 0.07	28.00 ± 0.27
1306_2	22.41 ± 2.48	0.60 ± 0.01	0.12 ± 0.02	0.03 ± 0.00	0.06 ± 0.03	5.19 ± 0.11	13.94 ± 1.24	174.74 ± 7.33	326.59 ± 3.96	3.26 ± 0.17	1.23 ± 0.12	0.86 ± 0.01	0.26 ± 0.06	11.32 ± 0.12
3306_1	55.59 ± 2.08	0.38 ± 0.03	0.04 ± 0.02	0.03 ± 0.00	0.09 ± 0.03	4.43 ± 0.04	26.13 ± 2.41	205.30 ± 8.68	371.69 ± 7.93	6.94 ± 0.19	0.13 ± 0.06	2.31 ± 0.08	0.12 ± 0.02	10.58 ± 0.55
3306_2	1960.58 ± 32.18	2.44 ± 0.08	0.57 ± 0.04	1.10 ± 0.03	1.19 ± 0.04	21.66 ± 0.42	332.50 ± 7.84	1849.62 ± 24.66	1960.60 ± 42.31	183.86 ± 2.07	2.06 ± 0.14	56.06 ± 0.41	3.42 ± 0.12	172.82 ± 1.75
1307	558.50 ± 21.21	0.44 ± 0.07	0.13 ± 0.03	0.31 ± 0.02	0.17 ± 0.03	4.98 ± 0.10	95.27 ± 1.56	491.12 ± 8.56	507.00 ± 16.74	32.42 ± 0.62	2.02 ± 0.11	7.12 ± 0.07	0.48 ± 0.05	55.84 ± 0.38
2307_1	3683.77 ± 39.42	1.83 ± 0.07	0.38 ± 0.05	1.48 ± 0.04	0.76 ± 0.02	17.41 ± 0.37	383.60 ± 8.77	1676.00 ± 29.44	1473.05 ± 33.05	239.23 ± 3.00	3.30 ± 0.22	37.62 ± 0.57	3.83 ± 0.12	124.30 ± 1.42
2307_2	708.02 ± 22.40	1.91 ± 0.11	1.03 ± 0.08	0.38 ± 0.04	0.91 ± 0.06	22.25 ± 0.42	346.28 ± 6.74	1595.81 ± 28.05	778.65 ± 27.35	55.52 ± 0.45	3.27 ± 0.12	53.01 ± 0.36	0.84 ± 0.11	210.85 ± 1.02
3307	170.22 ± 5.85	0.61 ± 0.08	0.09 ± 0.04	0.12 ± 0.01	0.23 ± 0.02	9.98 ± 0.22	71.48 ± 3.33	302.06 ± 9.71	437.08 ± 14.64	13.41 ± 0.16	0.67 ± 0.04	5.34 ± 0.17	0.25 ± 0.06	85.73 ± 1.26
1308_1	40.28 ± 2.82	0.65 ± 0.05	0.51 ± 0.05	0.07 ± 0.01	0.18 ± 0.03	7.40 ± 0.22	31.09 ± 2.59	816.64 ± 32.70	371.30 ± 8.83	3.96 ± 0.10	1.92 ± 0.15	7.08 ± 0.26	0.17 ± 0.03	53.05 ± 1.06
1308_2	37.68 ± 1.58	0.59 ± 0.06	0.44 ± 0.06	0.06 ± 0.02	0.11 ± 0.04	5.50 ± 0.10	27.87 ± 1.39	689.48 ± 13.16	350.33 ± 5.49	4.36 ± 0.17	1.41 ± 0.03	6.03 ± 0.05	0.25 ± 0.03	177.13 ± 2.08
2308_1	130.86 ± 6.22	0.62 ± 0.06	0.29 ± 0.05	0.09 ± 0.01	0.17 ± 0.03	10.25 ± 0.19	61.10 ± 2.60	395.44 ± 9.42	447.79 ± 15.79	12.97 ± 0.41	1.34 ± 0.07	7.98 ± 0.27	0.30 ± 0.04	41.60 ± 1.07

Sample ID	Al	As	Cd	Co	Cr	Cu	Fe	K	Mg	Mn	Ni	Pb	V	Zn
2308_2	2376.29 ± 23.85	2.18 ± 0.11	0.53 ± 0.05	1.58 ± 0.05	1.48 ± 0.06	23.92 ± 0.54	366.66 ± 10.10	2458.25 ± 56.37	1672.58 ± 22.38	305.44 ± 4.20	4.16 ± 0.12	51.65 ± 1.18	4.57 ± 0.19	247.10 ± 2.62
3308	1192.60 ± 9.13	0.93 ± 0.03	0.54 ± 0.04	0.77 ± 0.03	0.62 ± 0.04	17.62 ± 0.19	315.02 ± 6.46	1264.67 ± 9.50	1031.42 ± 19.73	94.65 ± 0.50	1.94 ± 0.07	27.88 ± 0.14	2.38 ± 0.09	165.57 ± 1.96
4308_1	21.49 ± 1.61	0.41 ± 0.05	0.04 ± 0.01	0.01 ± 0.01	0.07 ± 0.04	4.19 ± 0.07	20.78 ± 1.26	541.66 ± 6.11	279.91 ± 2.67	2.64 ± 0.11	0.12 ± 0.03	0.83 ± 0.00	0.03 ± 0.01	14.18 ± 0.17
4308_2	2099.81 ± 50.50	2.34 ± 0.11	0.71 ± 0.05	1.00 ± 0.03	1.69 ± 0.07	48.12 ± 0.09	797.68 ± 4.72	1495.61 ± 34.06	1702.59 ± 48.88	132.31 ± 0.96	5.27 ± 0.14	60.47 ± 0.35	4.64 ± 0.17	752.65 ± 8.11
1309_1	3299.98 ± 66.08	2.30 ± 0.09	0.43 ± 0.01	1.53 ± 0.07	1.51 ± 0.05	36.35 ± 0.36	597.82 ± 5.60	2552.15 ± 9.90	1971.17 ± 44.96	371.89 ± 5.21	5.29 ± 0.12	68.42 ± 0.43	4.43 ± 0.13	195.38 ± 1.86
1309_2	6262.73 ± 89.29	7.09 ± 0.12	2.66 ± 0.16	6.03 ± 0.10	4.99 ± 0.07	48.39 ± 0.49	3006.76 ± 34.09	6902.40 ± 234.50	6265.91 ± 92.59	953.32 ± 9.83	16.13 ± 0.23	693.28 ± 26.52	13.40 ± 0.67	1970.54 ± 21.43
2309_1	448.11 ± 25.60	1.64 ± 0.03	0.15 ± 0.04	0.27 ± 0.02	0.41 ± 0.05	13.48 ± 0.31	145.69 ± 4.02	1055.96 ± 20.09	605.57 ± 21.48	48.83 ± 0.76	2.37 ± 0.12	10.85 ± 0.38	0.88 ± 0.04	64.25 ± 0.75
2309_2	3690.30 ± 77.38	11.75 ± 0.37	0.83 ± 0.06	5.45 ± 0.08	2.72 ± 0.08	39.20 ± 0.50	738.63 ± 3.01	3665.97 ± 37.60	2790.82 ± 80.22	472.09 ± 3.78	5.90 ± 0.10	77.36 ± 0.51	6.75 ± 0.10	448.73 ± 1.48
3309	2083.41 ± 61.23	1.83 ± 0.03	0.38 ± 0.04	1.15 ± 0.04	0.67 ± 0.07	23.69 ± 0.54	384.36 ± 11.69	2277.60 ± 85.06	1228.28 ± 29.89	186.17 ± 2.93	2.09 ± 0.11	53.40 ± 1.94	3.31 ± 0.26	175.30 ± 3.64
4309	1101.71 ± 19.41	2.96 ± 0.03	0.37 ± 0.04	0.93 ± 0.04	0.93 ± 0.11	20.81 ± 0.27	270.11 ± 1.79	1537.75 ± 60.69	975.90 ± 20.61	78.50 ± 1.05	3.26 ± 0.09	22.48 ± 0.87	2.82 ± 0.17	120.57 ± 1.89
1310_1	2403.79 ± 53.28	1.56 ± 0.08	84.85 ± 3.64	1.73 ± 0.03	2.63 ± 0.06	47.63 ± 1.04	875.60 ± 20.09	3426.77 ± 105.80	2199.65 ± 33.93	225.67 ± 3.64	4.11 ± 0.16	48.86 ± 1.54	5.87 ± 0.48	644.57 ± 10.12
1310_2	2698.24 ± 46.47	1.32 ± 0.08	56.10 ± 2.56	1.92 ± 0.04	2.97 ± 0.08	32.35 ± 0.42	850.44 ± 9.86	3003.06 ± 95.34	2112.46 ± 53.80	202.92 ± 3.30	4.62 ± 0.09	44.68 ± 1.44	4.55 ± 0.24	764.81 ± 7.97

Sample ID	Al	As	Cd	Co	Cr	Cu	Fe	K	Mg	Mn	Ni	Pb	V	Zn
2310_1	22.66 ± 3.49	0.52 ± 0.04	0.06 ± 0.01	0.14 ± 0.03	0.09 ± 0.02	5.33 ± 0.15	16.63 ± 1.00	567.85 ± 6.52	365.57 ± 8.56	3.36 ± 0.16	0.27 ± 0.03	2.17 ± 0.03	-0.02 ± 0.03	25.23 ± 0.11
2310_2	1318.01 ± 31.92	1.00 ± 0.08	0.84 ± 0.03	0.55 ± 0.02	0.42 ± 0.02	13.96 ± 0.26	318.63 ± 3.17	767.80 ± 9.56	702.87 ± 13.14	74.25 ± 0.48	1.22 ± 0.04	37.16 ± 0.27	1.43 ± 0.07	121.35 ± 0.33
M3310_1	56.98 ± 3.30	0.44 ± 0.03	0.09 ± 0.00	0.16 ± 0.01	0.22 ± 0.03	7.51 ± 0.24	36.70 ± 1.51	358.96 ± 5.71	536.01 ± 12.73	4.41 ± 0.11	3.40 ± 0.11	1.26 ± 0.01	0.13 ± 0.02	28.99 ± 0.39
M2311	534.53 ± 12.17	2.47 ± 0.13	0.11 ± 0.03	1.09 ± 0.02	0.32 ± 0.03	39.11 ± 0.49	228.39 ± 3.21	852.50 ± 22.03	1002.66 ± 32.01	79.27 ± 0.45	1.37 ± 0.10	4.13 ± 0.04	1.60 ± 0.10	75.14 ± 0.61
M3310_2	3572.15 ± 68.54	4.24 ± 0.05	1.29 ± 0.05	7.13 ± 0.10	3.34 ± 0.18	49.17 ± 0.57	1178.49 ± 12.15	7838.62 ± 88.14	6976.88 ± 174.42	650.75 ± 5.56	10.60 ± 0.30	25.11 ± 0.24	15.27 ± 0.29	1100.77 ± 11.31
M3311_1	11979.19 ± 325.59	5.65 ± 0.16	4.25 ± 0.17	28.35 ± 0.43	8.78 ± 0.05	146.19 ± 1.61	2494.58 ± 33.17	15239.59 ± 261.57	17418.27 ± 425.88	2323.36 ± 26.44	50.42 ± 0.51	66.65 ± 0.24	35.07 ± 0.09	7030.91 ± 51.30
M3311_2	2077.95 ± 43.23	2.74 ± 0.08	1.83 ± 0.04	3.47 ± 0.07	2.24 ± 0.04	62.77 ± 0.47	777.98 ± 4.28	2675.04 ± 29.68	2590.51 ± 57.73	303.76 ± 3.37	7.79 ± 0.26	22.00 ± 0.17	10.51 ± 0.30	248.11 ± 1.72

Table S5. Indoor and outdoor $PM_{2.5}$ and PM_{10} summary with I/O Ratios.

Home ID	$PM_{2.5}$ Indoor (μg/m³)	$PM_{2.5}$ Outdoor (μg/m³)	PM_{10} Indoor (μg/m³)	PM_{10} Outdoor (μg/m³)	$PM_{2.5}$ I/O Ratio	PM_{10} I/O Ratio	ACH (hr^{-1})	$PM_{2.5}$ Decay Rate (hr^{-1})	PM_{10} Decay Rate (hr^{-1})
**2310**	2.47 ± 0.72	4.12 ± 1.69	8.07 ± 5.31	13.05 ± 3.04	0.60	0.62	12.37	---	---
**1310**	4.53 ± 0.44	1.99 ± 0.28	23.37 ± 11.04	14.15 ± 5.71	2.28	1.65	5.6	0.79	1.38
**4309**	3.67 ± 0.76	9.23 ± 1.54	26.75 ± 15.85	16.39 ± 4.43	0.40	1.63	2.56	---	---
**3309**	1.45 ± 0.28	1.85 ± 3.38	9.94 ± 8.75	6.21 ± 5.49	0.78	1.60	6.47	1.95	2.34
**2309**	3.05 ± 0.58	1.93 ± 0.25	26.15 ± 13.55	7.21 ± 3.52	1.58	3.63	2.38	---	---
**1309**	2.54 ± 0.83	2.67 ± 0.32	16.64 ± 9.54	9.35 ± 5.04	0.95	1.78	1.62	---	---
**4308**	10.74 ± 1.26	8.58 ± 1.38	75.71 ± 31.65	24.03 ± 5.97	1.25	3.15	0.43	0.99	1.9
**3308**	3.01 ± 0.51	9.12 ± 2.08	35.12 ± 17.76	17.89 ± 7.14	0.33	1.96	0.58	2.65	2.05
**2308**	2.24 ± 0.40	3.45 ± 0.62	23.91 ± 22.00	9.73 ± 4.76	0.65	2.46	0.31	0.75	2.27
**1308**	2.43 ± 0.62	4.53 ± 1.06	15.23 ± 8.23	15.43 ± 7.19	0.54	0.99	0.8	---	---
**3307**	1.28 ± 0.37	5.46 ± 45.11	11.33 ± 8.43	12.70 ± 45.70	0.23	0.89	0.42	---	---
**2307-2**	6.48 ± 1.08	2.54 ± 0.65	72.88 ± 34.85	9.65 ± 3.25	2.55	7.55	2.5	0.71	2.26
**2307-1**	5.44 ± 1.05	2.61 ± 0.50	54.70 ± 15.55	10.43 ± 4.12	2.08	5.24	2.5	---	---
**1307**	2.43 ± 0.34	7.72 ± 0.79	12.28 ± 6.77	12.67 ± 2.40	0.31	0.97	1.2	---	---
**3306**	2.87 ± 0.62	1.14 ± 0.55	82.19 ± 42.63	5.69 ± 4.70	2.51	14.44	0.073	1.63	0.92
**2306**	1.66 ± 0.26	3.30 ± 0.59	13.51 ± 7.51	20.17 ± 8.27	0.50	0.67	0.39	---	---
**1306**	2.37 ± 0.45	3.04 ± 0.46	30.39 ± 17.18	18.27 ± 8.08	0.78	1.66	0.26	---	---
**Average**	3.45 ± 2.33	4.31 ± 2.71	31.66 ± 24.45	13.12 ± 5.11	1.08 ± 0.81	2.99 ± 3.46	---	---	---

SUPPLEMENTARY FIGURES

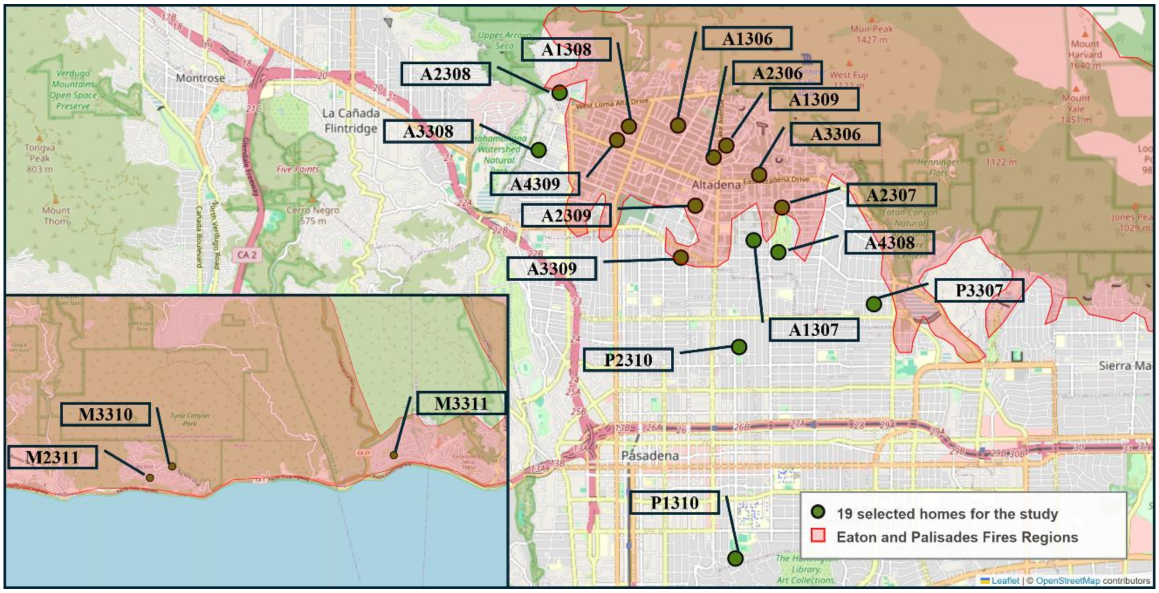

Figure S1. Eaton and Palisades Fires map with assigned labels to each sampled household.

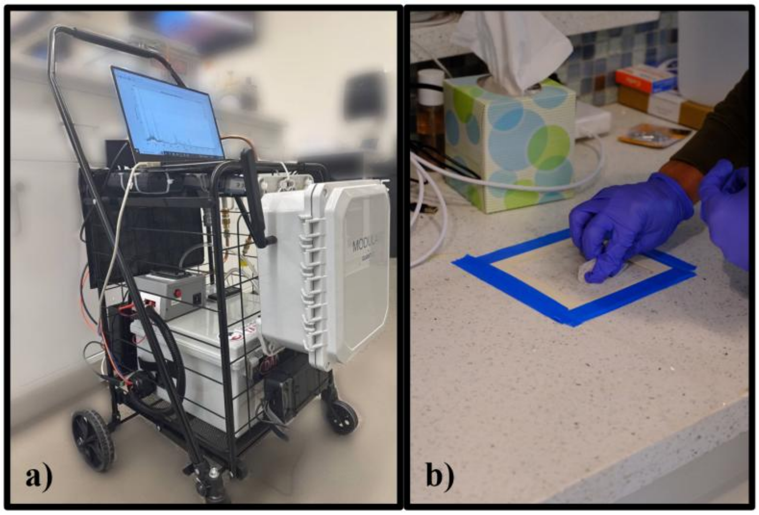

Figure S2. a) Sampling cart containing all the instruments with required batteries and b) Dust sample collection based on the EPA's method for lead dust wipe collection using a 10 × 10 cm² sampling template.

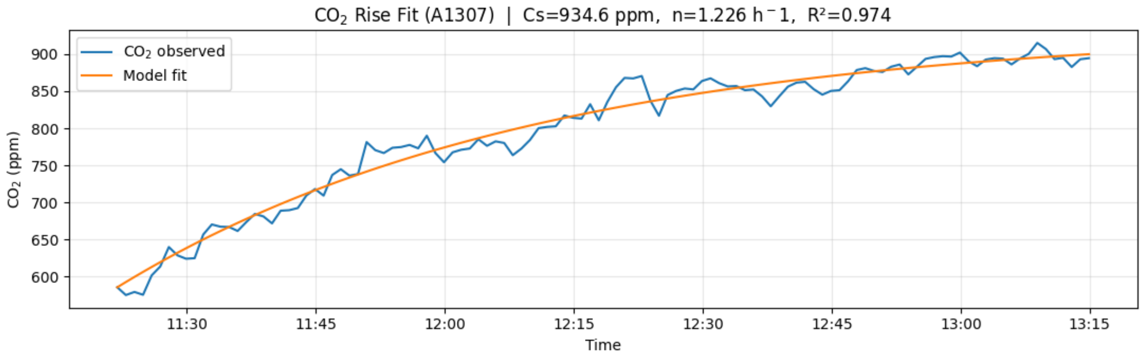

Figure S3. An example of the fitted model used to estimate the Css and ACH.

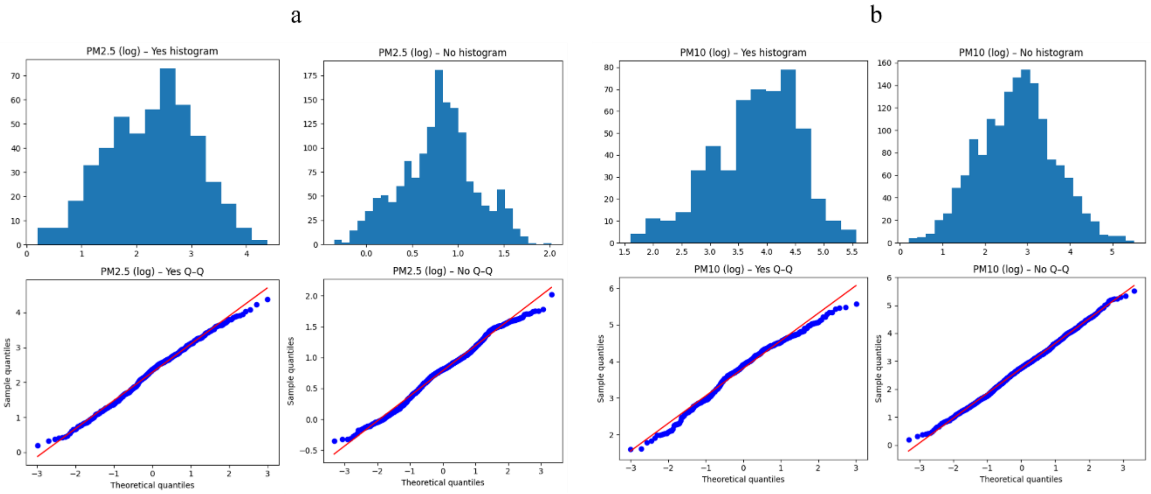

Figure S4. Histogram and Q-Q plots to check normality of log-transformed data for a) $PM_{2.5}$ and b) PM_{10}.

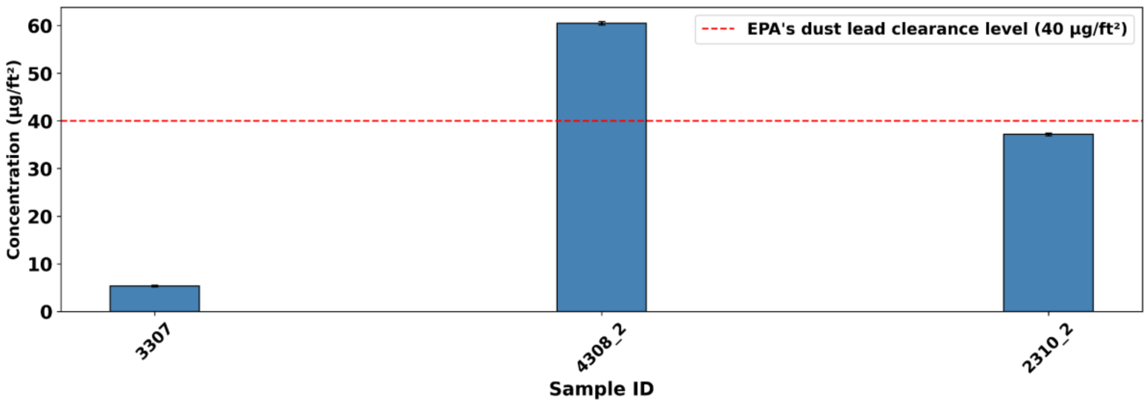

Figure S5. Lead concentrations in wipe samples taken from window sills.

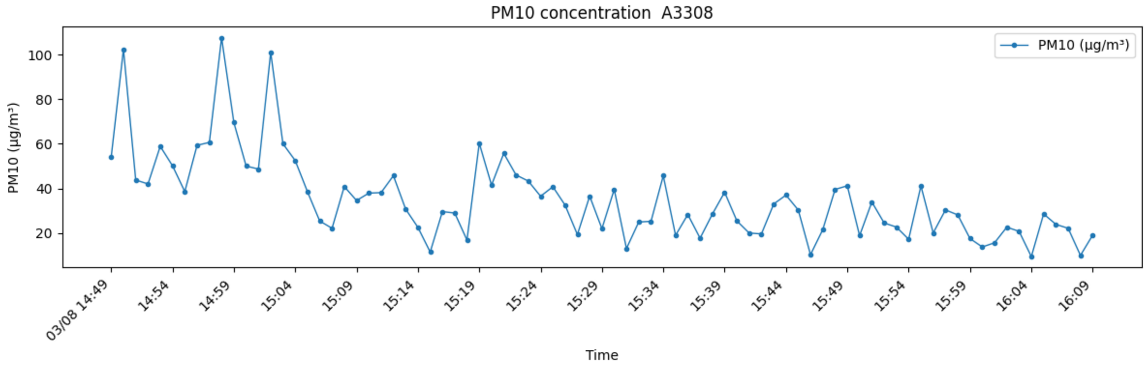

Figure S6. PM10 spikes during the first 15 minutes of indoor sampling (14:50 to 15:05), due to instrument setup, followed by a decay over time.

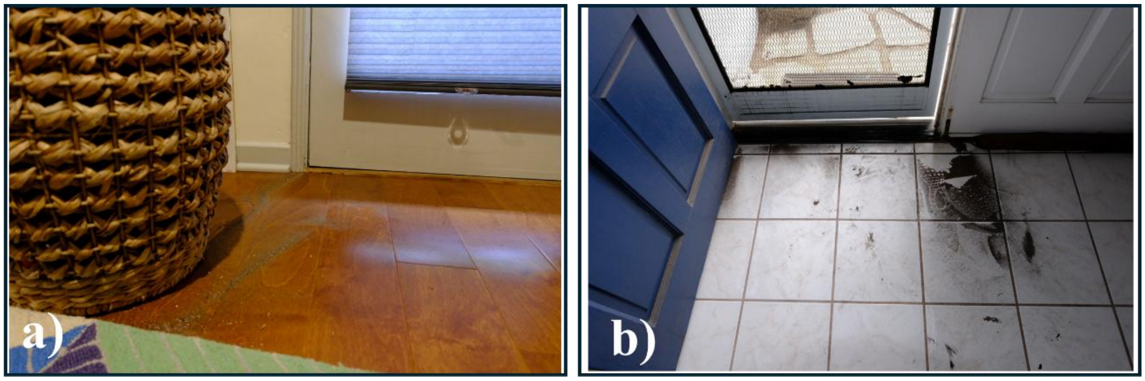

Figure S7. Surface contamination near the entry in a relatively a) low-contaminated and b) high-contaminated home.

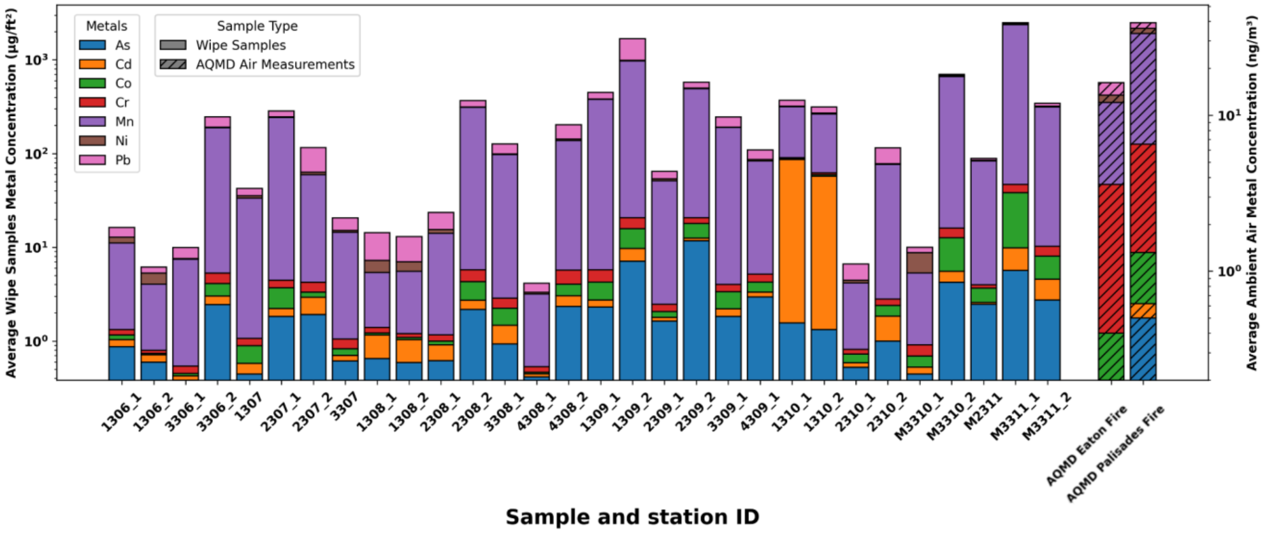

Figure S8. Stacked plot of average HAP metals concentrations in collected wipe samples from homes (left axis) and AQMD average HAP metals concentrations measured in ambient air (right axis). Note that the y-axis is on a log10 scale.
